# Digital health technologies and machine learning augment patient reported outcomes to remotely characterise rheumatoid arthritis

**DOI:** 10.1038/s41746-024-01013-y

**Published:** 2024-02-12

**Authors:** Andrew P. Creagh, Valentin Hamy, Hang Yuan, Gert Mertes, Ryan Tomlinson, Wen-Hung Chen, Rachel Williams, Christopher Llop, Christopher Yee, Mei Sheng Duh, Aiden Doherty, Luis Garcia-Gancedo, David A. Clifton

**Affiliations:** 1 Institute of Biomedical Engineering, Department of Engineering Science, University of Oxford, Oxford, UK; 2 Big Data Institute, University of Oxford, Oxford, UK; 3 Value Evidence and Outcomes (VEO), GSK, UK; 4 Nuffield Department of Population Health, University of Oxford, Oxford, UK; 5 Value Evidence and Outcomes (VEO), GSK, US; 6 Analysis Group (AG), Boston, MA USA

**Keywords:** Predictive markers, Rheumatoid arthritis, Fatigue, Chronic pain, Translational research

## Abstract

Digital measures of health status captured during daily life could greatly augment current in-clinic assessments for rheumatoid arthritis (RA), to enable better assessment of disease progression and impact. This work presents results from weaRAble-PRO, a 14-day observational study, which aimed to investigate how digital health technologies (DHT), such as smartphones and wearables, could augment patient reported outcomes (PRO) to determine RA status and severity in a study of 30 moderate-to-severe RA patients, compared to 30 matched healthy controls (HC). Sensor-based measures of health status, mobility, dexterity, fatigue, and other RA specific symptoms were extracted from daily iPhone guided tests (GT), as well as actigraphy and heart rate sensor data, which was passively recorded from patients’ Apple smartwatch continuously over the study duration. We subsequently developed a machine learning (ML) framework to distinguish RA status and to estimate RA severity. It was found that daily wearable sensor-outcomes robustly distinguished RA from HC participants (F1, 0.807). Furthermore, by day 7 of the study (half-way), a sufficient volume of data had been collected to reliably capture the characteristics of RA participants. In addition, we observed that the detection of RA severity levels could be improved by augmenting standard patient reported outcomes with sensor-based features (F1, 0.833) in comparison to using PRO assessments alone (F1, 0.759), and that the combination of modalities could reliability measure continuous RA severity, as determined by the clinician-assessed RAPID-3 score at baseline (*r*^2^, 0.692; RMSE, 1.33). The ability to measure the impact of the disease during daily life—through objective and remote digital outcomes—paves the way forward to enable the development of more patient-centric and personalised measurements for use in RA clinical trials.

## Introduction

Rheumatoid arthritis (RA) patients follow subtle and unpredictable disease courses, patient-to-patient, with a progressive decline in physical function and quality of life and over time—often leading to disability and difficulty to perform many tasks of daily life^1^. RA symptoms include joint pain or tenderness, joint swelling, morning stiffness, reduction in joint range of movement (ROM), muscle pain, and fatigue^1^. Currently, the gold-standard methods to measure the impact of RA on daily life rely on infrequent clinical visits that may often occur every 3–4 months, with assessments depending on a combination of subjective clinician-determined scores^2^ and patient-reported outcomes^3^. These have inherent limitations, however, in that they can be subjective and are prone to recall bias^4,5^. As such, there is a need to objectively measure the impact of RA on daily life^6^, remotely over a continuous period, rather than restricting assessments to only intermittent physician visits. In recent years, consumer-grade mobile applications (app.) and wearable devices have shown promise to objectively measure participants’ symptoms during daily life^7^; these digital health technologies (DHT) tools^8^ have shown to increase study engagement, improve patient convenience, streamline collection of PROs^9^, and potentially generate more frequent and accurate data that can characterise disease^10^. DHT have been shown to measure RA symptoms and functions, such as range of motion (ROM) and gait-specific metrics during prescribed “active” assessments^11,12^. Other studies have shown how “passive” wearable actigraphy sensor-outcome measurements capture differences in RA physical activity (PA) in daily life, compared to healthy controls (HC)^13^, as well as to detect flaring of RA symptoms^14^.

However, there remains a lack of sufficient evidence for how DHT can provide objective insights into the impact of therapies for RA, despite progress made in other disease areas^15–22^. Particularly, the benefit of sensor-outcomes generated from prescribed active assessments compared with passive monitoring has not yet been explored together. While digitised patient-reported outcomes (PROs) enable a patient the ability to regularly record their “subjective” experience of disease activity in remote settings^23^, it remains unclear how “objective” sensor-outcomes could provide additional insights that can augment PROs to better characterise the impact of RA on daily life. As part of this characterisation, the sensitivity of DHT to measure RA symptoms, such as the volume of remote data required and the number of sensor-outcome measurements needed, will also need to be determined. Finally, the application of DHT sensor-outcomes to monitor RA during daily life remains yet to be validated against standard in-clinic administered assessments of RA impact^24^.

In this study, we therefore aimed to investigate how active and passive sensor-based measurements should be combined using machine learning (ML) to distinguish RA status from healthy controls, to augment traditional patient self-reported outcome (PRO) data, and to estimate standard in-clinic assessments of RA severity. Our work offers the first comprehensive evaluation of how sensor data captured during daily life can characterise RA status and severity, which represents an important first step towards the development of more sensitive and patient-centric measurements for use in RA clinical trials and real-world studies.

In order to investigate the objectives of this study, we performed the following set of analysis and experiments. We first illustrate the variety of sensor-based measurements that can be extracted from daily prescribed (active) smartphone-based assessments and (passive) smartwatch-based activity monitoring in an RA cohort. In this, we evaluate how smartwatch-based daily physical activity patterns can be remotely estimated using our bespoke deep convolutional neural (DCNN), pre-trained using multi-task self-supervised learning (SSL) on a large-scale open-source cohort. We next assess the ability of our sensor-based measurements to identify RA status from healthy controls and to distinguish RA severity levels. As part of our analysis, we also explore the volume of days and number of sensor-outcomes required to remotely distinguish RA status. Finally, we investigated the power of active and passive sensor-outcomes to augment routinely collected patient self-reported outcome (PRO) data to estimate RA severity—as measured by standard in-clinic assessments of RA, such as the RAPID-3^25^.

## Results

**Fig. 1:**
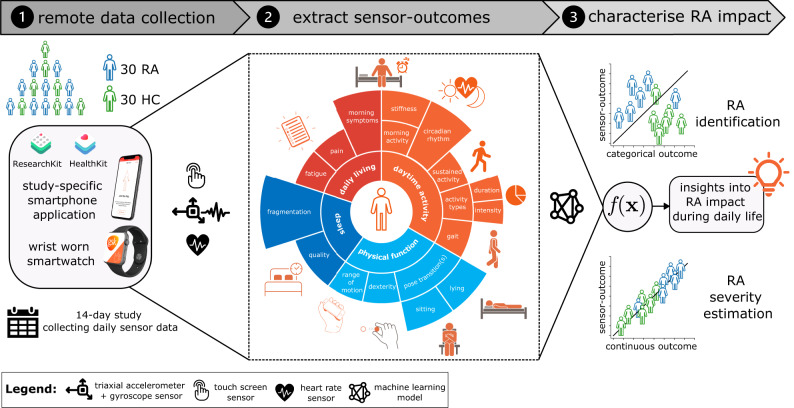
Illustration detailing the objectives of this study. The weaRAble-PRO 14-day trial aimed to investigate how digital health technologies (DHT)—a wrist-worn Apple smartwatch and an iPhone device, with bespoke mobile apps.—could augment patient reported outcomes (PRO) to characterise the impact of rheumatoid arthritis (RA) during the daily life of 30 moderate-to-severe RA patients, compared to 30 matched healthy controls (HC). We explore the ability of machine learning (ML) models to (1) estimate categorical RA outcomes, such as identifying RA participants from healthy controls and (2) estimate continuous RA outcomes, such as RA severity, using a combination of PRO and sensor-outcomes.

The GSK weaRAble-PRO study (GSK212295) was a 14-day observational study which investigated how DHT tools could objectively measure the impact of RA on participants’ daily lives. Digital wearable devices—a wrist-worn Apple Watch for passive monitoring and an iPhone, integrated with a bespoke mobile app. which prescribed daily guided assessments—collected high-frequency, objective sensor data in 30 RA patients and 30 matched Healthy Controls (HCs). Figure 1 provides an illustrative overview of the objectives of this study. Sensor-based measures of physical function, mobility, dexterity, and other RA specific symptoms were extracted from daily prescribed (active) iPhone guided tests using a combination of bespoke algorithms and proprietary algorithms developed by Apple ResearchKit, for instance, a wrist-range of motion exercise, a walking assessment, a nine-hole peg test, as well as two pose transition-based mobility exercises, lie-to-stand (LTS) and sit-to-stand (STS). In addition, continuous (passive) actigraphy was recorded from participants’ Apple smartwatch over the study duration in order to characterise daily activity patterns and sleep. In order to illustrate the various characteristics of RA we are interested in assessing, we have grouped measurements in Fig. 1 into four domains: physical function, daytime activity, daily living, and sleep; denoting particular types of measurements which may attribute to each domain. Note: this manuscript details a sub-study of weaRAble-PRO; trial design, feasibility, participant adherence, and other primary related study outcomes are reported in Hamy et al.^26^. Two RA participants withdrew immediately after enroling in the study. Data from these participants were not collected, leaving 28 RA participants, 28 matched HCs, and 2 unmatched HCs for a total of 58 participant

### Assessing smartwatch-based daily physical activity patterns

The daily physical activity of RA participants and healthy controls were estimated with a deep convolutional neural network (DCNN) that was first pre-trained on 100,000 participants in the publicly available UK Biobank, following a multi-task self-supervised learning (SSL) methodology^27^, which was subsequently fine-tuned on the free-living Capture-24 dataset^28^ of < 150 participants to determine broad activity patterns of interest {sleep, sedentary, light physical activity, moderate-to-vigorous physical activity (MVPA)}^29,30^ and fine-grained activity prediction labels {sleep, sitting/standing, mixed, vehicle, walking, bicycling}^28^. In this study, we build upon our previous work by adding a temporal dependency to the “DCNN (SSL)” through a hidden markov model (HMM), which was appended to obtain a more accurate sequence of predicted activities over the continuous study period. It was found that the “DCNN (SSL) + HMM” improved broad activity estimation in Capture-24 (*κ*, 0.862 ± 0.088; F1, 0.815 ± 0.103) as compared to a baseline random forest (RF) + HMM approach (*κ*, 0.813 ± 0.108; F1, 0.775 ± 0.117)^28^. Next, the fine-tuned “DCNN (SSL) + HMM” model transformed the raw Apple smartwatch sensor data in weaRAble-PRO to determine participants’ daily activity patterns over the 14-day study period, for example, the time spent walking, the frequency of exercise, the length and quality of sleep, and other RA-specific measures, such as morning stiffness. Activity predictions were qualitatively evaluated over the entire RA and HC study population and demonstrated face validity (see Supplementary Figs. 1 and 2 for additional details).

### Analysis of sensor-outcomes to distinguish RA status and severity levels

**Fig. 2:**
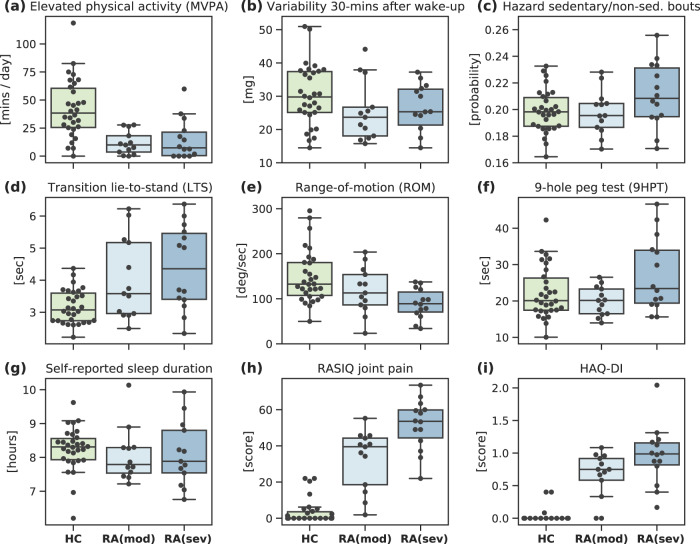
Ability of individual sensor-outcomes to distinguish between RA status and RA severity levels. Comparison of the average feature distributions per participants, between healthy controls (HC), RA (moderate) and RA (severe) groups for: **a**–**c** selection of passively collected smartwatch features; **d**–**f** selection of guided test collected smartphone features; and **g**–**i** selection of patient self-reported outcomes recorded on the smartphone application. For all examples shown, medians were significantly different between HC and RA groups: One-way ANOVA determined from the Kruskal-Wallis H-test, *p* < 0.001. deg degrees, HAQ-DI Health Assessment Questionnaire-Disability Index, min minutes, mg mili-gravity acceleration units, MVPA moderate-to-vigorous physical activity, RASIQ GSK RA symptom and impact questionnaire, sed sedentary, sec seconds.

The raw smartphone and smartwatch data recorded during the (active) guided test exercises, and passively during the participants’ daily life, respectively, were summarised as sensor-outcome features. Univariate analysis demonstrated that a total of 153 (93%) sensor-based features (passive, *n* = 131 (94%); active, *n* = 22 (88%)) displayed significantly different medians (after post-hoc correction for multiple comparisons) between HC and RA severity groups (Kruskal-Wallis H test, *p* < 0.05). A further 47 (34%) passive features, compared to 6 (24%) active features, were also significantly different (Mann-Whitney U test, *p* < 0.05) between healthy and RA participants. Figure 2 compares the (fortnightly) average feature distributions between healthy controls (HC), RA (moderate) and RA (severe) participants for a selection of examples of passively collected smartwatch features (Fig. 2a–c) and active guided test sensor features (Fig. 2d–f) and a selection of patient self-reported outcomes recorded on the smartphone application (Fig. 2g–i).

**Fig. 3:**
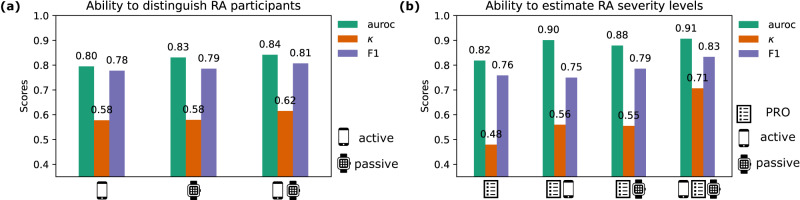
Ability of combined sensor-outcomes to distinguish between RA status and RA severity levels. Comparison of **a** RA identification (RA vs. HC) performance and **b** RA severity level estimation (RA (mod) vs RA (sev)), using patient reported outcomes (PRO) and combined PRO (list icon), active (smartphone icon), and passive (smartwatch icon) sensor-based outcomes in the weaRAble-PRO study. auroc area under the receiver operator curve, *κ* Cohen’s Kappa statistic, F_1_ macro-F1 score.

In order to explore the ability of many wearable sensor-outcomes to distinguish symptoms of RA from otherwise healthy individuals, and therefore measure the impact of RA during daily life, we devised a number of multivariate classification-based experiments. First, we investigated the performance of regularised logistic regression (LR) to differentiate RA participants from healthy controls using both passively collected activity monitoring features and guided test exercise features. Comparing model performance between sources (Fig. 3a), passive activity monitoring-based sensor features better distinguished RA participants using fortnightly averaged features (F1, 0.786) versus active (guided test) features (F1, 0.778). It was found that 12 subjects were misclassified using active-only models and 12 for passive-only, with just 4/12 (33%) of the same subjects incorrectly identified by both sources, 3 of which were the same HC participants. Combining active and passive wearable sensor features yielded in the highest performing models to distinguish RA participants overall, for example, using fortnightly averaged features from both sources (F1, 0.807) (for further expansion of results, see Supplementary Table 4). It should also be noted that linear logistic regression was found to perform comparatively to non-linear ensembles of decision trees, a Random Forest (RF) model and Extreme Gradient Boosted Trees (XGB)—as such this work subsequently opted to explore simple linear models for further analysis (see Supplementary Table 5).

This study next investigated the ability of multiple sensor-based outcomes to augment PRO data in order to stratify RA severity levels. In weaRAble-PRO, participants were denoted as having moderate or severe RA based on baseline clinician-assessed RAPID-3 scores. Following similar procedure to RA identification, LR regularised models were investigated in order to distinguish RA (mod) and RA (sev) as binary classification tasks using fortnightly averaged study data. The benefit of incorporating additional sensor-based outcomes to patient (self-) reported outcomes is presented in Fig. 3b (expanded in Supplementary Table 6). It was observed that the linear combination of PRO assessments could accurately stratify RA symptom severity (F1, 0.759). The fusion of PRO data and sensor-based outcomes improved RA severity level estimation further with the addition of active (F1, 0.750) or passive (F1, 0.786) sources. Finally, the amalgamation of PRO outcomes with both active and passive sensor-based outcomes resulted in the most accurate RA severity level estimation (F1, 0.833)—an improvement of 10% compared to PRO outcomes alone (Fig. 3b). For additional information on the selected PRO + sensor-outcomes, we refer the reader to Supplementary Table 3.

### Estimating the volume of days and number of sensor-outcomes required to remotely distinguish RA status

**Fig. 4:**
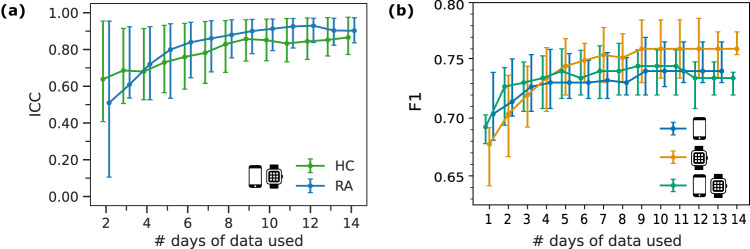
The number of days of sensor-data required to remotely characterise RA impact. Comparison of **a** the minimal amount of days of data needed distinguish RA status, as measured by the F1 score across 5-fold cross validation (CV), between active (smartphone icon), passive (smartwatch icon), and combined (smartphone & smartwatch icons) feature sources; **b** the feature (test-retest) reliability, as measured by the intraclass correlation coefficient (ICC), between RA participants and HC across the study duration (14 days); F1 scores and ICCs suggest that model performance and feature reliability stabilises once more than 7 days of data are used per participant.

In weaRAble-PRO, participants performed daily guided test exercises—resulting in daily sensor features—and continuously recorded Apple Watch sensor data were summarised as daily activity monitoring-based features, over the 14-day study period. In this work, we aimed to determine the minimal number of days of sensor data required build a stable and robust estimate of disease status in RA participants compared to HC over the 14-day study period. Figure 4a represents an experiment exploring the (observation-wise) out-of-sample RA classification performance as a function of varying the number of non-contiguous days of data that are averaged per participant. Evaluated over 500 randomly sampled permutations of non-contiguous days, results (median + IQR) indicated that RA prediction stabilised once more than 7 non-contiguous days of data were used per participant. Furthermore, we found that averaging daily feature values over weekly and fortnightly periods improved model performance. However, it was observed that model performance using weekly-averaged features was often similar to fortnightly averaged (we also refer the reader to Supplementary Table 4).

To investigate feature consistency and reproducibility, the intra-class correlation coefficient (ICC) for each feature was evaluated over the study duration (14 days). ICCs were calculated for each feature using *n* = [2, 3, …, 14] days of data per participant, individually for HC and RA participants. Higher ICC’s suggest a high degree of similarity on the performance of each task over the course of the study, and lower coefficients mean that participants tended to perform the task differently each day of the study. ICC’s for HCs ranged from 0.582 to 0.854, while those for RA participants ranged from 0.424 to 0.897. Figure 4b depicts the median + inter-quartile range (IQR) of ICC values for the LR-elastic net retained active + passive features. Intra-rater reliability analyses suggest that feature reliability stabilises to good (ICC=0.75–0.9) and excellent (ICC > 0.9) once more than 7 contiguous days of data were used per participant.

**Fig. 5:**
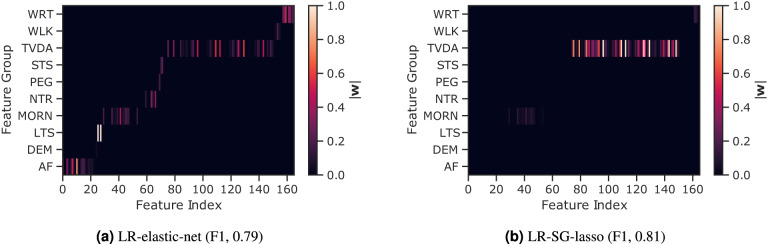
The number of sensor-outcomes required to remotely distinguish RA status. Comparison of features selected between regularised logistic regression (LR) models for: **a** elastic-net (F1, 0.79) and **b** SG-lasso (F1, 0.81). The SG-lasso promotes group-wise sparsity (i.e., regularising the number of feature domains) and within-group sparsity (i.e., regularising the number of features per domain), achieving a similar performance to LR elastic-net, while selecting a fewer number of domains and features. Feature importance, denoted as the mean LR coefficient value (w) over cross-validation, are illustrated by colour intensity. Feature domains: AF activity fragmentation, DEM demographics, LTS lie-to-stand assessment, MORN morning stiffness, NTR night-time restlessness, PEG 9-hole peg test, STS sit-to-stand assessment, TVDA total volume of daytime activity, WLK walking assessment, WRT wrist assessment.

In order to evaluate the number of sensor-outcomes required to remotely distinguish RA status, we compared various feature regularisation techniques, lasso (*ℓ*_1_), ridge (*ℓ*_2_), elastic-net (*ℓ*_1_+*ℓ*_2_), and sparse-group lasso, using fortnightly (i.e., study duration) averaged features. It was found that introducing sparsity through regularisation improved classification performance. In addition, active and passively recorded sensor-based features could be grouped into domains, based on the guided test they were extracted from, or the perceived functional domain of daily activity they were assumed to assess. Introducing group-wise sparsity with the sparse-group lasso (SG-lasso), regularising on the number of groups (i.e., the feature domains) and the coefficients within each group, resulted in the highest RA participant identification performance (F1, 0.807), compared to lasso (*ℓ*_1_, F1, 0.772), ridge (*ℓ*_2_, F1, 0.792), and elastic net (*ℓ*_1_+*ℓ*_2_, F1, 0.792) regularisation (for expansion of results, see Supplementary Table 5). The features and groups selected by each regularisation technique are illustrated in Fig. 5, represented as the mean LR coefficient value **w** over CV per each feature and feature domain (coefficient values have been normalised between 0 and 1 to benefit comparison between models). Examining the feature sparsity of elastic-net (*ℓ*_1_ + *ℓ*_2_) (Fig. 5a), it was observed that features from multiple domains were selected. In contrast, the SG-lasso, as shown in Fig. 5b, selected mostly passive activity-based smartwatch features—TVDA with some morning stiffness measures—to distinguish RA status. Group sparsity penalised simultaneously selecting from multiple feature domains, where within group-sparsity regularised the feature coefficient values within the selected domains. Using fewer domains and less features, the SG-lasso was able achieve similar performance to LR elastic-net, even marginally improving performance (F1, 0.807). For further details on the features extracted, and selected, we refer the reader to the Supplementary Methods.

### Estimating in-clinic RA severity scores from PRO and sensor-based outcomes

Rheumatoid arthritis severity levels were denoted by a clinician administered RAPID-3 assessment^25^ at baseline in the weaRAble-PRO study. The RAPID-3—a “rapid” and easy to administer questionnaire—is also validated against more exhaustive assessments for RA, such as the disease activity score 28 (DAS28) and clinical disease activity index (CDAI) in clinical trials and clinical care^25^. In this work, we aimed to establish how the combination of PRO and sensor-based outcomes could stratify continuous RAPID-3 RA severity. Note: HC subjects were assigned a RAPID-3 score of zero at baseline. Through multivariate modelling, using LR elastic-net, it was determined that PRO and sensor-based features could accurately estimate RAPID-3 scores to within 1 point (*r*^2^, 0.69; MAE, 0.94; RMSE, 1.33), an improvement compared to using PRO measures alone (*r*^2^, 0.63; MAE, 1.16; RMSE, 1.45). The association between actual and PRO + sensor-outcome estimated RAPID-3 scores was found to be good-to-excellent (*r* > 0.75), Pearson’s r = 0.60, *p* < 0.001; Spearman’s *ρ* = 0.83, *p* < 0.001.

**Fig. 6:**
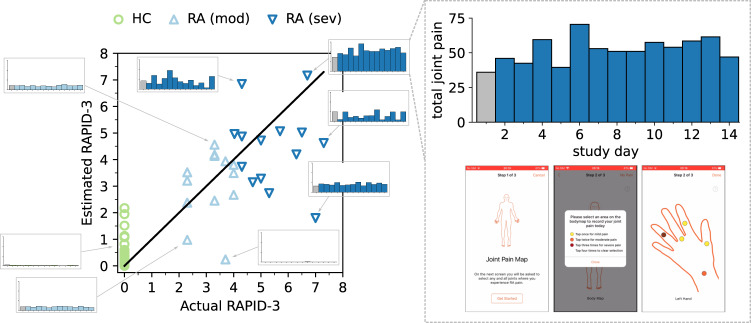
The ability of remote PRO + sensor-outcomes to estimate in-clinic determined RA severity scores. Scatter plot of baseline RAPID-3 scores *y* versus predicted $\hat{y}$ scores per subject, using elastic net with PRO + sensor-outcomes, over cross-validation (CV). Participant model-estimated RAPID-3 scores can be further interpreted through detailed inspection of the daily smartphone-based patient-reported joint pain map (JMAP) total scores—which was not included as a predictor in the model. Higher JMAP scores indicate higher levels of pain experienced. Additional interpretability, through the JMAP, demonstrated that PRO + sensor-based outcome estimation of the RAPID-3 could reliably reflect patient’s perceived daily RA symptoms. Note: Baseline JMAP total scores, recorded on the same day as the baseline RAPID-3, are denoted in grey; the JMAP y-axis scale is the same among all subplots. HC subjects were assigned a RAPID-3 score of zero at baseline. A black line represents perfect predictions (*r*^2^, 0.692; MAE, 0.938; RMSE, 1.333).

Participants in weaRAble-PRO were also administered a twice-daily interactive Joint Pain Map (JMAP) questionnaire on their iPhone^11^, in order to more precisely record and localise perceived pain. Participant model-estimated RAPID-3 scores were further interpreted through detailed inspection of the daily smartphone-based patient-reported joint pain map (JMAP) total scores—an external validation measure, which was not included as a predictor in the model—as expanded in Fig. 6. The JMAP score, defined as the sum of all individual joint pain scores per recording, was intended as a coarse measure to holistically capture participants’ overall level of perceived pain, in addition to validated PRO assessments. Higher JMAP scores indicate higher levels of pain experienced. It was observed that RAPID-3 estimations were reliable and robust, in that they faithfully characterised RA participant’s perceived level of symptoms, through the JMAP. For example, in Fig. 6, the RA (sev.) participant with consistently the largest reported degree of pain across the 14-day study exhibited the highest actual RAPID-3 score (6.7), which was closely estimated by the model at 7.1. JMAP scores further enabled additional explanation of model performance, especially with respect to RAPID-3 estimations that were not reflective of actual RAPID-3 scores. For instance, the RA (mod) participant with the lowest estimated RAPID-3 score (0.2) actually reported zero pain experienced over the 14-day study duration, despite a RAPID-3 assignment of 3.7 at baseline. Non-zero estimated RAPID-3 scores for some HC could also often be contextualised, due to these participants frequently self-reporting low-levels of pain in their JMAP (i.e., non-zero JMAP entries) over the study period, despite being healthy. As such, it was determined that PRO and sensor-based RAPID-3 estimates could reliably reflect participant’s RA symptoms over the study.

## Discussion

Our findings in the weaRAble-PRO study demonstrate how digital health technology (DHT) captured sensor-outcomes, recorded from smartphone-based active tests, and continuously collected passive smartwatch-based monitoring, could characterise meaningful aspects of rheumatoid arthritis (RA) impairment and physical function impacting daily life. Remotely collected wearable sensor-outcomes could distinguish RA status from healthy controls—demonstrating further improved performance when combining the sensor-data from both devices—and how objective sensor-outcomes could augment patient (self-) reported outcomes to remotely estimate RA severity. Furthermore, by the half-way point of the weaRAble-PRO study (day 7), a sufficient volume of data had already been collected to reliably distinguish the characteristics of RA participants. This work provides the first comprehensive evaluation how remote and objective digital sensor-outcomes enrich our ability to understand the impact of RA on daily life between clinical visits.

In this work, we detailed how raw data collected from smartphone and smartwatch sensors can be transformed into sensor-based outcomes that are reflective of disease status. In concurrence with previous studies, many remotely collected smartphone sensor-outcomes distinguished RA participants and RA severity levels. For example, it was observed that joint ROM features differentiated HC and RA groups—a similar finding to our previous work^12^—and that RA participants were less mobile, taking longer to move between positions (as measured during the lie-to-stand exercise)—as previously shown by Andreu-Perez et al.^31^. Continuously collected smartwatch sensor data, known as passive monitoring, allowed the measurement of aspects of RA daily life, such as physical activity, sleep, and other RA specific symptoms, such as morning stiffness, or night-time restlessness. In this study we trained an activity recognition model on the free-living capture-24 dataset to estimate daily activity patterns in the wearable-pro population. Leveraging the latest advances in self-supervised learning (SSL) allowed our model to be pre-trained on 100,000 participants with 700,000 days of diverse, unlabelled wearable sensor data in the uk biobank^27^, which combined with HMM temporal smoothing, significantly improved activity prediction compared to our previous established RF-HMM based methods^28,30^. Our SSL DCNN+HMM model enabled a more robust and fine-grained estimation of daily activity patterns beyond traditional acceleration magnitude levels^13,14^, which we proposed could allow a richer characterisation of PA and sleep in RA activity monitoring revealed distinct differences distinguishing RA status, for example the daily percent of the day in moderate-to-vigorous physical activity, and similar features, were significantly lower in the RA population compared to healthy controls—a similar finding by Prioreschi et al.^13^, and an observation people with RA regularly self-report ^32^. Other specific RA symptom measurements, like morning stiffness or disrupted sleep, were evident in certain RA participants. For example, the mean acceleration value > 30 [mins] after wake-up were lower in RA—also a similar finding to Keogh et al.^33^—or that the number of movement episodes during night-time sleep distinguished some specific RA participants. We also observed that after collecting 7 days of sensor-data in the weaRAble-PRO study, a sufficient volume of data had already been recorded to reliably distinguish RA participants from a healthy population; participant feature reliability (as measured ICC values) stabilised at good-to-excellent levels, maximal identification performance of RA participants plateaued, and that there was no additional benefit to averaging over a fortnight’s worth of data versus a week. Therefore it is recommended that considering at least one week’s worth of sensor data is collected, it might be more beneficial to gather less data from a greater number of participants, rather than greater duration of sensor data from the same participants.

Our work is the first study to combine active smartphone and passive wearable measurements to distinguish RA status and measure variations in RA severity. While models trained on only passive features tended to marginally outperform models trained solely on active guided test features, combining both active + passive features led to the best performance in RA identification for all models investigated. Interestingly, it was found that different subjects were misclassified by active versus passive models. For example, 12 subjects were misclassified using active-only models and 12 for passive-only, with just 4/12 (33%) of the same subjects incorrectly identified by both sources, 3 of which were the same HC participants. In addition, further experiments with the LR-SG-lasso determined that only activity monitoring domain features were mainly needed in order to distinguish RA participants from health controls. This indicates that we sometimes do not need to prescribe all guided test assessments, or to parse all activity feature domains, but that a small number of prescribed assessments can be sufficient to characterise RA status. For example, including only the lie-to-stand assessment rather than also prescribing the similar, and highly correlated, sit-to-stand assessment in future studies; or removing the prescribed walking assessment (shown to have little predictive value in the weaRAble-PRO study), and using passive daily life walking predictions generated from the activity recognition model instead, which could reduce patient burden. Finally, we also found that combining patient-reported outcomes (PRO) and objective sensor-outcomes could better capture RAPID-3-based RA severity at baseline than PROs alone; most estimated RAPID-3 scores correctly stratified participants across severity levels from healthy to moderate to severe RA, suggesting that sufficient information to characterise RA disease severity could be reflected in the remote monitoring outcomes derived in the 14-day weaRAble-PRO study. To the best of the authors knowledge, this offers the first evaluation and insight how remote monitoring outcomes in daily life can estimate in-clinic administered assessments of RA impact.

There are a number of limitations that must be considered in the weaRAble-PRO study. Despite rich individual level measurements, the study recruited a relatively small sample size (HC, *n* = 30; RA, *n* = 30). As such, a degree of variability and uncertainty existed in constructing cross-validated models to distinguish RA participants, RA severity levels, or estimate the in-clinic RAPID-3 assessment. Extrapolation of results aimed at generalising RA is therefore not possible without the availability of larger cohorts and further external validation. In addition, this study only recruited RA patients with moderate-to-severe levels of disease activity; future studies should also aim to characterise patients with lower levels of disease activity or those in remission. There were also limitations associated with modelling a clinician-administered assessment, or clinical labels formulated from in-clinic assessments. For instance, the RAPID-3 was assessed at baseline, with participants recalling the prior week, yet the PRO and sensor-based features were calculated as averages over subsequent 14-day trial period from baseline. As such, the baseline RAPID-3 may not have precisely reflected the participant’s disease status recorded earlier, due to the underlying mutability and heterogeneity of RA symptoms over short periods of time. The subjectivity of PRO predictors should also considered, for instance, pain or perceived quality of sleep is relative, and some healthy participants recorded experiencing pain or affected sleep in PRO questionnaires. As a result, some PRO values influenced HC RAPID-3 predictions greater than zero, i.e., indicating the presence of RA symptoms—albeit non-zero estimated RAPID-3 predictions for HCs were generally low ( < 2).

The weaRAble-PRO study typifies how continuously collected patient self-reported and sensor-based outcomes may more closely reflect participant perceived and experienced symptoms that impact daily life. While in-clinic assessments are considered the gold-standard means of assessing disease severity in RA, it is clear that remotely collected, continuous, patient-centric measurements generated from PRO and sensor-based outcomes offer promising insights that can undoubtedly augment in-clinic assessments for RA. We believe that our work—the first comprehensive evaluation how remote sensor data can augment traditional PRO measures to estimate clinician-determined RA severity—helps informs future DHT study design to better characterise the impact of RA on daily life, ultimately to expand the use of DHT to develop more sensitive, and patient-centric, endpoints in RA clinical trials and real-world studies.

## Methods

### Dataset

Remotely collected smartphone and smartwatch sensor data was obtained from the GSK study title: Novel Digital Technologies for the Assessment of Objective Measures and Patient Reported Outcomes in Rheumatoid Arthritis Patients: A Pilot Study Using a Wrist-Worn Device and Bespoke Mobile App. (212295, weaRAble-PRO)^26^. This observational study followed 30 participants diagnosed with moderate-to-severe RA and 30 matched HCs over 14 days. The population demographics, in-clinic, and relevant patient self-reported outcomes, as assessed at baseline, are reported in Table 1. RA participants were denoted as displaying moderate disability, RA (mod), or severe disability, RA (sev), as determined by their baseline RAPID-3 score. Note: Two RA participants withdrew immediately after enroling in the study. Data from these participants were not collected, leaving 28 RA participants, 28 matched HCs, and 2 unmatched HCs for a total of 58 participants. All study information, informed consent, study questions and instructions for conducting the guided tests were first drafted in the form of a survey instrument. The survey instrument was then programmed into the mobile app. All documentation including the study protocol, any amendments, and informed consent procedures, were reviewed and approved by Reliant Medical Group’s IRB. All participants provided written informed consent before any study procedures were undertaken. The study was conducted in accordance with the International Committee for Harmonisation principles of Good Clinical Practice and the Declaration of Helsinki. We refer the reader to Hamy et al.^26^ for further study details. In addition, participant requirement and data collection are outlined in the accompanying Supplementary Methods material.

**Table 1:** Population demographics, in-clinic, and selected patient self-reported outcomes, as assessed at baseline, where the mean ± standard deviation across the population is reported.

	HC^a^	RA (mod)^b^	RA (sev)^c^	*p*^1^
	(*n* = 28)	(*n* = 13)	(*n* = 15)	
**Demographics**				
Age, years	58.4 ± 9.9	56.9 ± 11.4	60.4 ± 7.1	0.33
Female, n (%)	25 (89%)	11 (84%)	14 (93%)	0.92
BMI	25.8 ± 4.6	31.1 ± 5.9	31.7 ± 8.6	0.96
**In-clinic Outcome(s)**				
RAPID-3	0 ± 0	3.2 ± 0.7	5.3 ± 1.1	< 0.001
**Patient Reported Outcome(s)**				
HAQ-DI	0 ± 0	0.63 ± 0.36	1.03 ± 0.42	< 0.01
RASIQ-pain	3.1 ± 6.7	32.1 ± 20.8	56.2 ± 11.6	< 0.01
RASIQ-stiffness	5.9 ± 9.5	33.9 ± 18.9	51.6 ± 10.2	< 0.05
RASIQ-impact	47.3 ± 5.0	53.9 ± 5.1	50.8 ± 7.6	0.33
FACIT	49.2 ± 2.9	38.9 ± 4.3	31.9 ± 7.6	< 0.05
PROMIS-sleep	49.6 ± 2.8	52.7 ± 4.2	52.4 ± 4.3	0.83
PROMIS-pain	42.2 ± 4.8	54.2 ± 7.29	58.8 ± 4.6	0.09
JMAP total pain^2^	0.20 ± 0.5	13.5 ± 13.9	18.8 ± 13.7	0.23

^1^*p*, *p*-value calculated from Mann Whitney U-test comparing severe vs. moderate RA participants;

^2^*Note:* self-reported JMAP is not a validated PRO in RA;

^a^Matched HC to RA participants only;

^b^RA participants with baseline RAPID-3: 6.1–12.

^c^RA participants with baseline RAPID-3: > 12;

#### Sensor-based data collection

The Apple Watch and iPhone were used to collect high frequency raw sensor data from predefined, (active) guided tests on a daily basis. Participants were prescribed daily to perform five iPhone-based assessments: WRT, a wrist range of motion (ROM) exercise^12^; WLK, a 30-second walking exercise^12^; PEG, a digital 9-hole peg test^34^; STS, a sit-to-stand transition exercise^31,35^; and LTS, a lie-to-stand transition exercise^31,35^. A brief overview of the guided tests prescribed in weaRAble-PRO are presented in Supplementary Table 8. In addition, the Apple Watch was used to continuously collect background sensor data (denoted passive data), as the participants went about their daily activities. Participants were asked to maintain a charge on both the Apple Watch and the iPhone, so that interruptions to monitoring and data transfer were kept to a minimum. Since night-time activity was also monitored, while participants were asleep, it was requested that charging should be done during the day, in a way that fit the participants’ schedules (e.g., charging in the morning while getting ready for the day). For more details on the activity monitoring features, see Supplementary Table 9.

#### Patient-reported outcomes

Patient-reported outcomes (PRO), most often self-report questionnaires, were administered to assess disease activity, symptoms, and health status and quality of life from the patients’ perspective^36,37^. The weaRAble-PRO study administered a selection of validated PRO measures for RA in complement to bespoke digital PRO assessments—that are validated in clinical trials, where the questions, response options, and the general approach to assessment were standardised for all participants. PROs were recorded on days 1, 7, and 14 of data collection. The PRO assessments administered to participants are outlined in Supplementary Table 7.

### Smartwatch-based estimation of daily life patterns

**Fig. 7:**
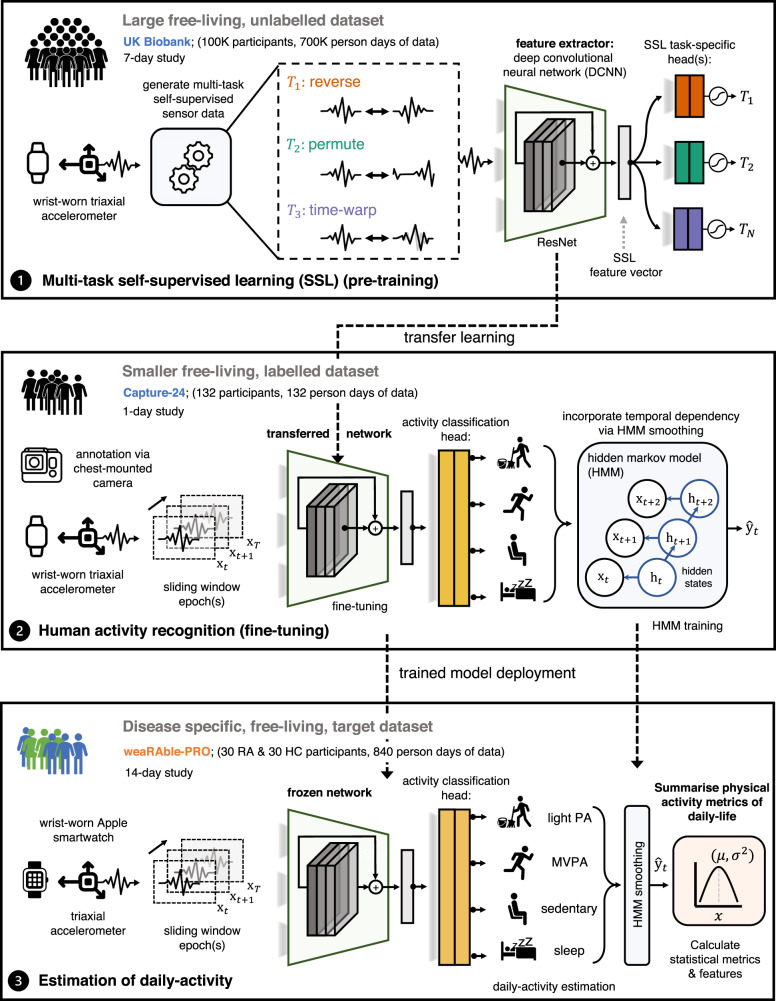
Self-supervised learning pipeline. Continuous (passive) actigraphy was recorded from patients' Apple smartwatch over the study duration. Deep convolutional neural networks (DCNN) were pre-trained on 700,000 person days in the publicly available UK Biobank using self-supervised learning—and fine-tuned with the Capture-24 dataset—to estimate participant’s daily activity patterns in the weaRAble-PRO study. Physical activity (PA) metrics of daily-life, for example, the time spent walking, the frequency of exercise, or the length and quality of sleep were investigated as markers to characterise symptoms of disease in people with RA compared to HC.

In order to generate unobtrusive measures characterising physical activity and sleep in RA participants during daily life, the raw Apple Watch actigraphy (i.e., accelerometer) sensor data was transformed through a human activity recognition (HAR) sensor processing and deep convolutional neural network (DCNN) pipeline. Figure 7 illustrates how a deep convolutional neural network (DCNN) can transform raw Apple smartwatch sensor data to estimate a participant’s daily activity patterns in the weaRAble-PRO study using self-supervised learning (SSL). The construction of this pipeline yielded unobtrusively measured summary features of physical activity and sleep for RA participants, computed daily during normal life.

A deep convolutional neural network (DCNN) with a ResNet-V2 architecture was first pre-trained following a multi-task self-supervised learning (SSL) methodology on 100,000 participants, each participant contributing 7 days yielding roughly 700,000 person days of data, in the open-source UK biobank^27^. The SSL pre-trained model was then fine-tuned to perform activity recognition as a downstream task in the Capture-24 dataset.

The Capture-24 study is a manually labelled, free-living dataset—that is reflective of real-world environments—and is available for training an activity recognition model to be applied to the weaRAble-PRO study. In Capture-24, actigraphy data was collected for 24-h from 132 healthy volunteer participants with a Axivity AX3 wrist-worn device as they went their normal day. Activity labels provided by photographs automatically captured roughly every 30 seconds by a wearable camera for each participant. Capture-24 was labelled with 213 activity labels, standardised from the compendium of physical activities^29^. Activity labels were then summarised into a small number of free-living behaviour labels, defining activity classes in Capture-24.

There are two major labelling conventions used within Capture-24 that the model was trained to predict, defined as broad activity: {sleep, sedentary, light physical activity, moderate-to-vigorous physical activity (MVPA)}^29,30^; and fine-grained activity: {sleep, sitting/standing, mixed, vehicle, walking, bicycling}^28^.

HAR model predictions are essentially independent—meaning that the sequence of activities over each 30 s epoch incorporates no temporal information epoch-to-epoch, for instance how the previous epoch prediction affects the current, or next, activity prediction. In order to add temporal dependency to the “DCNN (SSL)” model, a Hidden Markov Model (HMM) was implemented in a post-processing step to obtain a more accurate sequence of predicted activities over the continuous 14-day data collection period as per Willetts, et al.^28^.

This Capture-24 fine-tuned “DCNN (SSL) + HMM” model was then implemented to estimate daily activities in weaRAble-PRO study data. For additional information of the HAR deep network, SSL, and other related information, we refer the reader to our previous work^27^. Further results relating to the “DCNN (SSL)” models are outlined in the Supplementary Table 1. The sensor processing pipeline developed for the Apple Watch in the weaRAble-PRO study is outlined in Supplementary Fig. 5 and within the accompanying Supplementary Methods.

### Extraction of sensor-based outcomes

Wearable sensor-based features were derived from the smartphone during the active guided tasks and passively from the smartwatch during daily life. “Active” features, extracted from smartphone sensor-based measurements during the prescribed guided tests, aimed to capture specific aspects of RA physical function, related to pain, dexterity, mobility and fatigue^12^. In addition “passive” features were extracted from smartwatch sensor-based measurements, collected continuously in the background over the 14-day period. Daily activity predictions from the ML SSL model were summarised into general features measuring activity levels, period, duration and type of activity, as well as sleep detection and sleeping patterns. Furthermore, devised under the guidance of Rheumatologists, additional activity monitoring features specifically aimed at characterising well-known RA symptoms were also developed, such as morning stiffness and night-time restlessness.

The Supplementary Methods also detail algorithms used to extract active and passive features in the weaRAble-PRO study. For a full list of extracted sensor-based features in weaRAble-PRO, we refer the reader to Supplementary Table 9.

### Statistical analysis

#### Univariate testing

Pair-wise differences groups between groups, for example HC vs. RA, or RA (mod) vs. RA (sev) were analysed for the equality in population median using the non-parametric Mann-Whitney U test (MWUT)^38–40^. One-way analysis of variance (ANOVA) tests were also used to assess differences between medians of multiple groups, for example HC vs. RA (mod) vs. RA (sev) were assessed using the Kruskal-Wallis (KWt) test by ranks^41^. The Brown-Forsythe (BF) test by (absolute deviation) of medians was used to investigate if various groups of data have been drawn with equal variances^42^.

#### Correlation analysis

Correlation analysis was utilised to determine the association or dependence between sets of random variables, such as the dependence between features, or to assess a features’ clinical utility by measuring the association to an established clinical metric. This study investigated the (linear) Pearson’s *r* correlation and the (non-linear) Spearman’s Rho *ρ* correlation between features, between features and PROs, and between clinical assessments to determine levels of association. The strengths of the correlations were classified as good-to-excellent (*r* > 0.75), moderate-to-good (*r* = 0.50–0.75), fair (*r* = 0.25–0.49) or no correlation (*r* < 0.25)^43^.

#### Feature reliability

Intra-rater (i.e., test-retest) reliability was determined using intra-class correlation coefficient (ICC) values^44^, which were used to assess the degree of similarity between repeated features over the course of the study for each patient. In this work, the *ICC*(3, *k*) was calculated^45^–which considers the two-way random average measures with *k* repeated measurements—for the 14-day session across subjects, where the raters *k* are the study days. Reliability was categorised as either poor (ICC < 0.5), moderate (ICC=0.5–0.75), good (ICC=0.75–0.9), or excellent (ICC > 0.9)^46^.

#### Correcting for multiple hypothesis testing

Multiple hypothesis testing was performed due to the large volume of features by controlling the false discovery rate (FDR) at level *α* using the linear step-up procedure introduced by Benjamini and Hochberg (BH)^47,48^.

### Machine-learning estimation of RA status and severity

This work explored how state-of-the art machine learning (ML) models characterise the impact of RA during the daily life of participants in the 14-day weaRAble-PRO study. Multivariate modelling aimed to explore the ability of active, passive, and PRO measures to (1) distinguish RA participants from healthy controls (HC), and (2) to estimate RA disease severity: between RA participants with moderate symptoms (RA mod) and severe symptoms (RA sev) as binary classification tasks. Expansions of this analysis subsequently investigated how the in-clinic RAPID-3 assessment, a continuous measure of RA severity, could be estimated from the combination of PRO and sensor-based outcomes.

#### Overview of models

This analysis compared both linear and non-linear ML models to transform PRO and sensor-based outcomes to capture RA status and severity. Regularised linear regression (LR) models, with combinations of *ℓ*_1_ and *ℓ*_2_ priors, such as LR-lasso (*ℓ*_1_), LR-ridge (*ℓ*_2_), and LR-elastic-net (*ℓ*_1_ +*ℓ*_2_) were compared to yield predictive, yet sparse model solutions^49^. Further regularisation extensions were also investigated using the sparse-group lasso (SG-lasso)—an extension of the lasso that promotes both group sparsity and within group parameter-wise (*ℓ*_2_) sparsity, through a group lasso penalty and the lasso penalty—which aims to yield a sparse set of groups and also a sparse set of covariates in each selected group^50,51^.

Linear regression regularised models were also compared to decision tree (DT) based non-linear models, for instance the off-the-shelf Random Forest (RF)^52^ and Extreme Gradient Boosted Trees (XGB)^53^. Both LR- and DT-based models can intrinsically perform regression or classification depending on the task required. In the LR case, classification is denoted as logistic regression (though a logit-link function). NOTE: in this analysis LR can refer to both linear regression for continuous outputs or logistic regression for classification outputs. In the DT case, the mean prediction of the individual trees creates a continuous output for regression. For further details on the models employed in this study, we refer the reader to the Supplementary Methods.

#### Model evaluation

To determine the generalisability of our models, a stratified subject-wise k-fold cross-validation (CV) was employed. This consisted of randomly partitioning the dataset into k=5 folds, which was stratified with equal class proportions where possible. Participant data remained independent between training, validation, and testing splits. One set was denoted the training set (in-sample), and the remaining 20% of the dataset was then denoted testing set (out-of-sample) on which predictions were made.

#### Feature-wise and prediction-wise aggregation

In this work, we experimented with feature-wise and prediction-wise aggregation. In feature-wise aggregation, features were computed either as: daily feature values over the 14-day study period; the average daily feature value over a 7-day period (weekly); the average daily feature value over a 14-day period (fortnightly). Predictions could then be evaluated for each day (denoted *observation-wise*) or aggregated over all days through majority voting each individual prediction per subject (denoted *subject-wise*). For example, daily and weekly averaged features result in daily, or weekly predictions (i.e., *observation-wise*), which were summarised into *subject-wise* outcomes by majority voting over the repeated predictions.

#### Evaluation metrics

Multi-class classification metrics were reported as the *observation-wise* median and interquartile (IQR) range over one CV, as well as the *subject-wise* outcome for that CV, using: auroc, area under the receiver operating characteristic curve; *k*, Cohen’s kappa statistic^54,55^; F_1_, F1-score. The coefficient of determination, *r*^2^, the mean absolute error (MAE), and root-mean squared error (RMSE) were used to evaluate modelling the (continuous) in-clinic RAPID-3 scores^56^.

### Supplementary information

Supplementary Material Rendered

## Data Availability

Anonymised individual participant data that support the findings of this study are available from the corresponding author, upon reasonable request and subject to GSK’s approval.

## Code availability

Apple Watch sensor processing was performed using a bespoke version of the biobankAccelerometerAnalysis toolkit, found at: https://github.com/OxWearables/biobankAccelerometerAnalysis. Deep networks were built using Python v3.7 through a PyTorch v1.7 framework. Our self-supervised learning activity prediction code and trained models are publicly available at: https://github.com/OxWearables/ssl-wearables, including pre-trained models on 100K participants in the UK Biobank. Some guided test exercises and health metrics calculated are proprietary to Apple ResearchKit (http://researchkit.org/) and Apple HealthKit (https://developer.apple.com/documentation/healthkit) which we refer the reader for more details. Statistical and machine learning analysis was developed using scikit-learn v1.1.1. Further analysis code can be made available from the corresponding author upon reasonable request.

## References

[1] Grassi W, De Angelis R, Lamanna G, Cervini C (1998). The clinical features of rheumatoid arthritis. Eur. J. Radiol..

[2] Banderas B, Skup M, Shields AL, Mazar I, Ganguli A (2017). Development of the rheumatoid arthritis symptom questionnaire (rasq): a patient reported outcome scale for measuring symptoms of rheumatoid arthritis. Curr. Med. Res. Opin..

[3] Lubeck DP (2004). Patient-reported outcomes and their role in the assessment of rheumatoid arthritis. Pharmacoeconomics.

[4] Campbell, R., Ju, A., King, M. T. & Rutherford, C. Perceived benefits and limitations of using patient-reported outcome measures in clinical practice with individual patients: a systematic review of qualitative studies. *Quality Life Res.* 1–24 (2021). 10.1007/s11136-021-03003-z 34580822

[5] Gossec L, Dougados M, Dixon W (2015). Patient-reported outcomes as end points in clinical trials in rheumatoid arthritis. RMD Open.

[6] Flurey CA, Morris M, Richards P, Hughes R, Hewlett S (2014). It’s like a juggling act: rheumatoid arthritis patient perspectives on daily life and flare while on current treatment regimes. Rheumatology.

[7] Piga, M., Cangemi, I., Mathieu, A. & Cauli, A. Telemedicine for patients with rheumatic diseases: systematic review and proposal for research agenda. *In Seminars in Arthritis and Rheumatism*, Vol. 47, 121–128 (Elsevier, 2017). 10.1016/j.semarthrit.2017.03.014 28420491

[8] Taylor KI, Staunton H, Lipsmeier F, Nobbs D, Lindemann M (2020). Outcome measures based on digital health technology sensor data: data-and patient-centric approaches. NPJ Digital Med..

[9] Yun H (2020). Assessing rheumatoid arthritis disease activity with patient-reported outcomes measurement information system measures using digital technology. Arthritis Care Res..

[10] Munos B (2016). Mobile health: the power of wearables, sensors, and apps to transform clinical trials. Ann. New York Acad. Sci..

[11] Crouthamel M (2018). Using a researchkit smartphone app to collect rheumatoid arthritis symptoms from real-world participants: feasibility study. JMIR mHealth uHealth.

[12] Hamy V (2020). Developing smartphone-based objective assessments of physical function in rheumatoid arthritis patients: the PARADE study. Digital Biomarkers.

[13] Prioreschi A, Hodkinson B, Avidon I, Tikly M, McVeigh JA (2013). The clinical utility of accelerometry in patients with rheumatoid arthritis. Rheumatology.

[14] Gossec L (2019). Detection of flares by decrease in physical activity, collected using wearable activity trackers in rheumatoid arthritis or axial spondyloarthritis: an application of machine learning analyses in rheumatology. Arthritis Care Res..

[15] Pourahmadi MR (2017). Reliability and concurrent validity of a new iphone® goniometric application for measuring active wrist range of motion: a cross-sectional study in asymptomatic subjects. J. Anatom..

[16] Pratap A (2020). Evaluating the utility of smartphone-based sensor assessments in persons with multiple sclerosis in the real-world using an app (elevateMS): observational, prospective pilot digital health study. JMIR mHealth uHealth.

[17] Webster, D. E. et al. Clinical validation of digital biomarkers and machine learning models for remote measurement of psoriasis and psoriatic arthritis. *medRxiv* (2022).

[18] Omberg L (2022). Remote smartphone monitoring of Parkinson’s disease and individual response to therapy. Nat. Biotechnol..

[19] Creagh AP (2021). Smartphone- and smartwatch-based remote characterisation of ambulation in multiple sclerosis during the two-minute walk test. IEEE J. Biomed. Health Inf..

[20] Creagh A (2020). Smartphone-based remote assessment of upper extremity function for multiple sclerosis using the draw a shape test. Physiol. Measur..

[21] Lipsmeier F (2022). Reliability and validity of the Roche PD mobile application for remote monitoring of early parkinson’s disease. Sci. Rep..

[22] Lipsmeier F (2022). A remote digital monitoring platform to assess cognitive and motor symptoms in huntington disease: cross-sectional validation study. J. Med. Internet Res..

[23] El Miedany Y (2016). Toward electronic health recording: evaluation of electronic patient-reported outcome measures system for remote monitoring of early rheumatoid arthritis. J. Rheumatol..

[24] Coravos A, Khozin S, Mandl KD (2019). Developing and adopting safe and effective digital biomarkers to improve patient outcomes. NPJ Digital Med..

[25] Pincus T, Yazici Y, Bergman MJ (2009). Rapid3, an index to assess and monitor patients with rheumatoid arthritis, without formal joint counts: similar results to das28 and cdai in clinical trials and clinical care. Rheum. Dis. Clin..

[26] Hamy V (2023). Patient-centric assessment of rheumatoid arthritis using a smartwatch and bespoke mobile app in a clinical setting. Sci. Rep..

[27] Yuan, H. et al. Self-supervised learning for human activity recognition using 700,000 person-days of wearable data. *arXiv preprint arXiv:2206.02909* (2022). 10.1038/s41746-024-01062-3 PMC11015005 38609437

[28] Willetts M, Hollowell S, Aslett L, Holmes C, Doherty A (2018). Statistical machine learning of sleep and physical activity phenotypes from sensor data in 96,220 uk biobank participants. Scientific reports.

[29] Ainsworth BE (2011). 2011 compendium of physical activities: a second update of codes and met values. Med. Sci. Sports Exerc..

[30] Walmsley, R. et al. Reallocating time from device-measured sleep, sedentary behaviour or light physical activity to moderate-to-vigorous physical activity is associated with lower cardiovascular disease risk. *MedRxiv* (2020).

[31] Andreu-Perez J (2017). Developing fine-grained actigraphies for rheumatoid arthritis patients from a single accelerometer using machine learning. Sensors.

[32] Sokka T (2008). Physical inactivity in patients with rheumatoid arthritis: data from twenty-one countries in a cross-sectional, international study. Arthritis Care & Research: Official Journal of the American College of Rheumatology.

[33] Keogh A (2020). A thorough examination of morning activity patterns in adults with arthritis and healthy controls using actigraphy data. Digital Biomarkers.

[34] Mathiowetz V, Weber K, Kashman N, Volland G (1985). Adult norms for the nine hole peg test of finger dexterity. The Occupational Therapy Journal of Research.

[35] Bohannon RW (1995). Sit-to-stand test for measuring performance of lower extremity muscles. Perceptual and motor skills.

[36] of Health UD (2006). Guidance for industry: patient-reported outcome measures: use in medical product development to support labeling claims: draft guidance. Health and Quality of Life Outcomes.

[37] Mercieca-Bebber R, King MT, Calvert MJ, Stockler MR, Friedlander M (2018). The importance of patient-reported outcomes in clinical trials and strategies for future optimization. Patient Related Outcome Measures.

[38] Mann, H. B. & Whitney, D. R. On a test of whether one of two random variables is stochastically larger than the other. The Annals of Mathematical Statistics 50-60 (1947).

[39] Hollander, M., Wolfe, D. A. & Chicken, E. *Nonparametric statistical methods*, Vol. 751 (John Wiley & Sons, 2013).

[40] Gibbons, J. D. & Chakraborti, S. Nonparametric Statistical Inference: Revised and Expanded (CRC press, 2014).

[41] Kruskal WH, Wallis WA (1952). Use of ranks in one-criterion variance analysis. J. Am. Stat. Assoc..

[42] Brown MB, Forsythe AB (1974). Robust tests for the equality of variances. J. Am. Stat. Assoc..

[43] Portney, L. G. & Watkins, M. P. Foundations of clinical research: applications to practice, vol. 892 (Pearson/Prentice Hall Upper Saddle River, NJ, 2009).

[44] Weir JP (2005). Quantifying test-retest reliability using the intraclass correlation coefficient and the sem. J. Strength Condit. Res..

[45] Shrout PE, Fleiss JL (1979). Intraclass correlations: uses in assessing rater reliability. Psychol. Bull..

[46] Koo TK, Li MY (2016). A guideline of selecting and reporting intraclass correlation coefficients for reliability research. J. Chiropr. Med..

[47] Shaffer JP (1995). Multiple hypothesis testing. Ann. Rev. Psychol..

[48] Benjamini Y, Hochberg Y (1995). Controlling the false discovery rate: a practical and powerful approach to multiple testing. J. R. Stat. Soc.: Ser. B (Methodological).

[49] Hastie, T., Tibshirani, R. & Friedman, J.The elements of statistical learning: data mining, inference, and prediction (Springer Science & Business Media, 2009).

[50] Friedman, J., Hastie, T. & Tibshirani, R. A note on the group lasso and a sparse group lasso. *arXiv preprint arXiv:1001.0736* (2010).

[51] Simon N, Friedman J, Hastie T, Tibshirani R (2013). A sparse-group lasso. J. Comput. Graph. Stat..

[52] Breiman L (2001). Random forests. Mach. Learn..

[53] Chen, T. & Guestrin, C. Xgboost: A scalable tree boosting system. In *Proceedings of the 22nd acm sigkdd international conference on knowledge discovery and data mining*, 785–794 (2016).

[54] He H, Garcia EA (2009). Learning from imbalanced data. IEEE Trans. Knowl. Data Eng..

[55] Cohen J (1960). A coefficient of agreement for nominal scales. Educ. Psychol. Meas..

[56] Rao, C. R. Linear statistical inference and its applications, vol. 2 (Wiley New York, 1973).

