# Supplementary material for: Digital health technologies and machine learning augment patient reported outcomes to remotely characterise rheumatoid arthritis
Source: NPJ Digit Med. 2024 Feb 12;7:33. doi: 10.1038/s41746-024-01013-y (PMC10861520; doi:10.1038/s41746-024-01013-y)
Supplement: Supplementary file 1 — Supplementary Material Rendered [file 41746_2024_1013_MOESM1_ESM.pdf]

Digital health technologies and machine learning augment patient reported outcomes to remotely characterise rheumatoid arthritis

Andrew P. Creagh[*1,2], **Valentin Hamy**[3], **Hang Yuan**[2,4], **Gert Mertes**[1,2,4], **Ryan Tomlinson**[5], **Wen-Hung Chen**[5], **Rachel Williams**[5], **Christopher Llop**[6], **Christopher Yee**[6], **Mei Sheng Duh**[6], **Aiden Doherty**[†,2,4], **Luis Garcia-Gancedo**[†,3], **and David A. Clifton**[†,1]

[1]Institute of Biomedical Engineering, Department of Engineering Science, University of Oxford, UK;
[2]Big Data Institute, University of Oxford, UK;
[3]Value Evidence and Outcomes (VEO), GSK, UK;
[4]Nuffield Department of Population Health, University of Oxford, UK;
[5]Value Evidence and Outcomes (VEO), GSK, US;
[6]Analysis Group (AG), Boston, MA, USA.

ABSTRACT

Digital measures of health status captured during daily life could greatly augment current in-clinic assessments for rheumatoid arthritis (RA), to enable better assessment of disease progression and impact. This work presents results from weaRAble-PRO, a 14-day observational study, which aimed to investigate how digital health technologies (DHT), such as smartphones and wearables, could augment patient reported outcomes (PRO) to determine RA status and severity in a study of 30 moderate-to-severe RA patients, compared to 30 matched healthy controls (HC). Sensor-based measures of health status, mobility, dexterity, fatigue, and other RA specific symptoms were extracted from daily iPhone guided tests (GT), as well as actigraphy and heart rate sensor data, which was passively recorded from patients' Apple smartwatch continuously over the study duration. We subsequently developed a machine learning (ML) framework to distinguish RA status and to estimate RA severity. It was found that daily wearable sensor-outcomes robustly distinguished RA from HC participants (F1, 0.807). Furthermore, by day 7 of the study (half-way), a sufficient volume of data had been collected to reliably capture the characteristics of RA participants. In addition, we observed that the detection of RA severity levels could be improved by augmenting standard patient reported outcomes with sensor-based features (F1, 0.833) in comparison to using PRO assessments alone (F1, 0.759), and that the combination of modalities could reliability measure continuous RA severity, as determined by the clinician-assessed RAPID-3 score at baseline (r^2, 0.692; RMSE, 1.33). The ability to measure the impact of disease during daily life—through objective and remote digital outcomes—paves the way forward to enable the development of more patient-centric and personalised measurements for use in RA clinical trials.

*Corresponding author: andrew.creagh@eng.ox.ac.uk;
†These authors jointly supervised.

Supplementary Results

Assessing smartwatch-based daily physical activity patterns Supplementary Figure 1a–1b summarises the population-wide daily activity by time of the day for HC and RA groups. The probability of an activity being performed at a specific time can be computed as the number of instances detected for that activity across all data points (participants and days) at that time divided by the total number of data points. Representative examples of the predicted daily activity patterns for an individual healthy control (HC) and RA (moderate) participant are depicted in Supplementary Fig. 1c–1d respectively. The times when the Apple Watch was left to charge can be clearly seen in each example, indicated by the white non-wear times, typically occurring after wake-up or before bedtime. Both participants demonstrated consistent wake-up and bed times, day-to-day—which the activity prediction model tended to correctly identify.

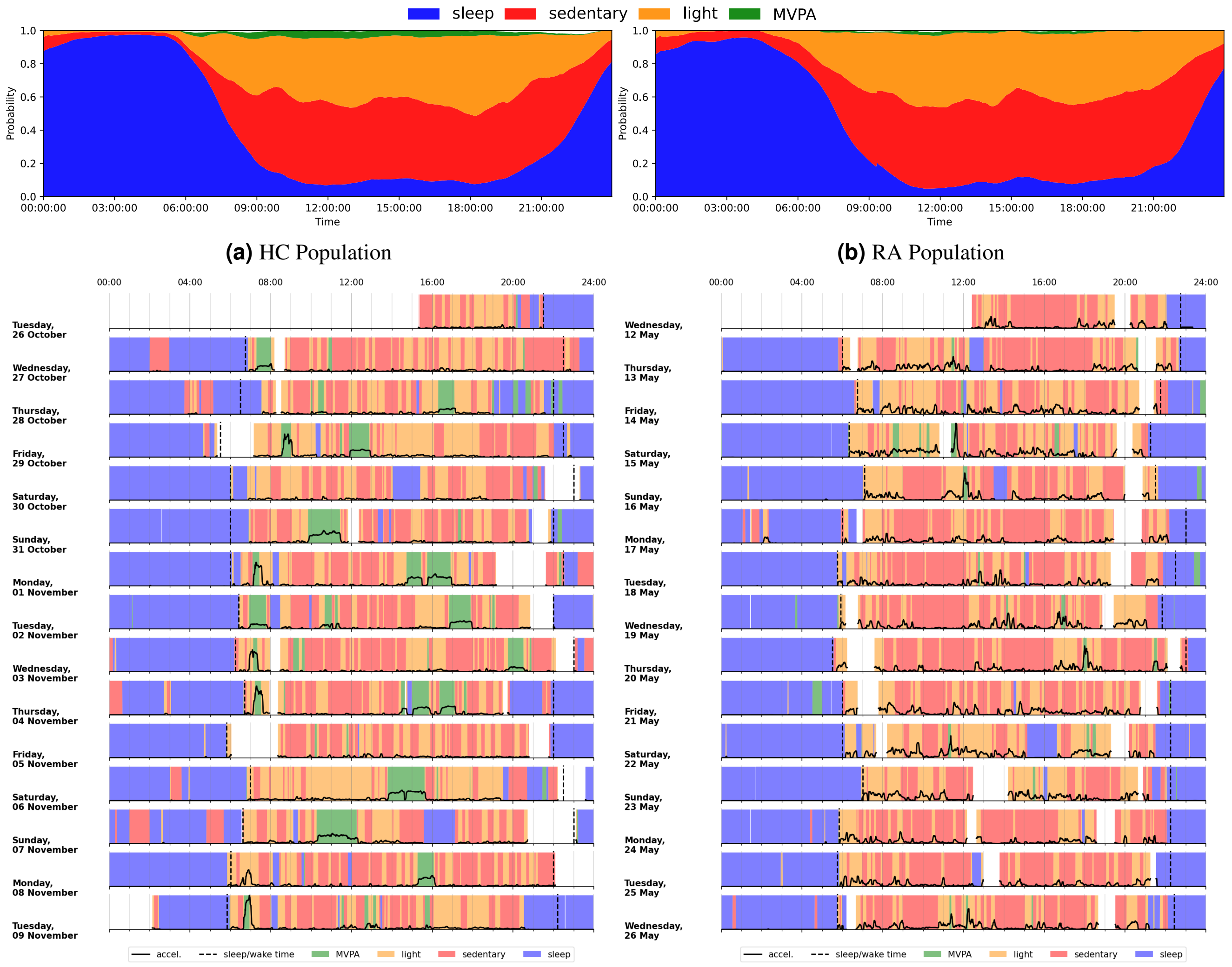

(a) HC Population

(b) RA Population

(c) HC participant

(d) RA (mod) participant

Supplementary Figure 1. Assessing smartwatch-based daily physical activity patterns. Variation in the average predicted daily-activity (probability) over time for all (a) HC participants and (b) RA participants in the 14-day weaRAble-PRO study. Predicted daily activity patterns for an individual (c) healthy control (HC) participant, (Female, 66 yrs.) and (d) a moderate Rheumatoid Arthritis (RA mod) participant (Female, 50 yrs.; RAPID-3, 3.7). Moving average acceleration values are overlaid in black. Participant self-reported sleep / wake times are indicated with long-dashed black lines. Non-wear times (expected daily for watch charging) are indicated by white areas. Note: the acceleration y-axis scaling between (c) and (d) is not the same due to difference in the magnitude of acceleration between participants. MVPA, moderate-to-vigorous physical activity.

Supplementary Table 1. Comparison of activity recognition performance in the Capture-24 dataset between baseline random forest (RF) model and ResNet-based deep convoutional neural network (DCNN), pre-trained on 700,000 person days in the UK Biobank following a self-supervised learning (SSL) framework. κ, Cohen's kappa statistic; F_1, macro-F1 score.

model	κ	f1
RF	0.705 ± 0.103	0.704 ± 0.102
RF + HMM	0.813 ± 0.108	0.775 ± 0.117
DCNN (SSL)	0.760 ± 0.087	0.735 ± 0.091
DCNN (SSL) + HMM	$\mathbf{0.862} \pm \mathbf{0.088}$	$\mathbf{0.815} \pm \mathbf{0.103}$

[*] HMM: hidden markov model posterior smoothing, see section for more details.

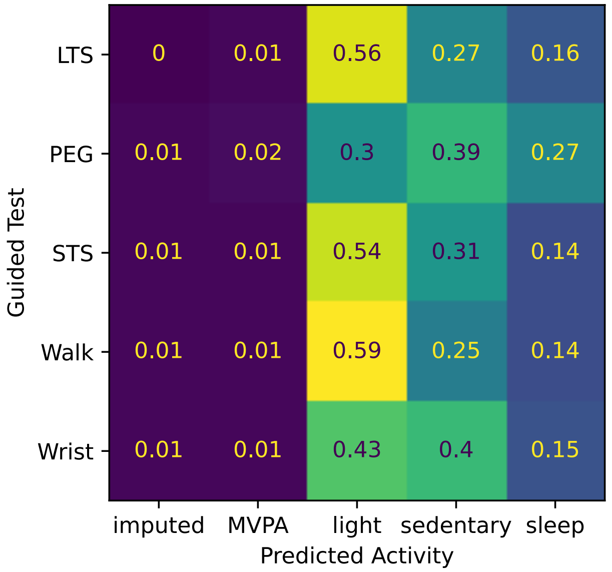

Supplementary Figure 2. Validation of activity predictions in the weaRAble-PRO study. Normalised confusion matrix evaluation of the "DCNN (SSL) + HMM" human activity recognition model on the weaRAble-PRO study with guided test timings as pseudo-labels.

Evaluation of activity recognition model The performance of the SSL model, compared to a feature-based Random Forest (RF) as a baseline, is reported in table 1. It was observed that the SSL model improved activity recognition performance in Capture-24 beyond feature-based approaches and training a model end-to-end. Furthermore, an insight into the performance of the best performing model, "DCNN (SSL) + HMM", in the weaRAble-PRO dataset was obtained from a set of simple experiments. While the Capture-24 study uses a similar device (Axivity AX3) and placement (non-dominant wrist) to the weaRAble-PRO's Apple Watch, the evaluation of the HAR predictions are unknown due to the lack of activity labels in the weaRAble-PRO study. However, as participant's performed prescribed guided tests during the day, HAR predictions could be bench-marked against the timing of these assessments. As such, guided test timings can act as pseudo-labels in order to evaluate the "DCNN (SSL) + HMM" model's robustness in applied to the weaRAble-PRO study, shown in Supplementary Fig. 2. As expected, during activity-based guided test assessments, such as walking or sit-to-stand and lie-to-stand, the HAR model more often predicts that participants are performing light activity. Guided assessments that require participants to be stationary while performing the task, such as the PEG test or wrist ROM test, are predicted more as sedentary activities. The percentage of sleep-based predictions during the guided test assessments (although incorrect) are roughly in line with the overall probability of daytime sleep, as observed depicted in figures 1c–1d. Further work is needed to fully characterise and appraise the predictions of daytime sleep, which are assumed to be incorrect predictions of sedentary activity.

Assessing feature dependence The relationship between the wearable sensor-based features extracted in this study, for both active (smartphone) and passive (smartwatch) data sources were investigated using pair-wise Spearman's ρ correlation. Correlation analysis indicated good-to-excellent relationships ($\rho > 0.75$) between many features within feature domains (intra-source); for example, most TVDA features were highly correlated with each other (positively and negatively). Analysis

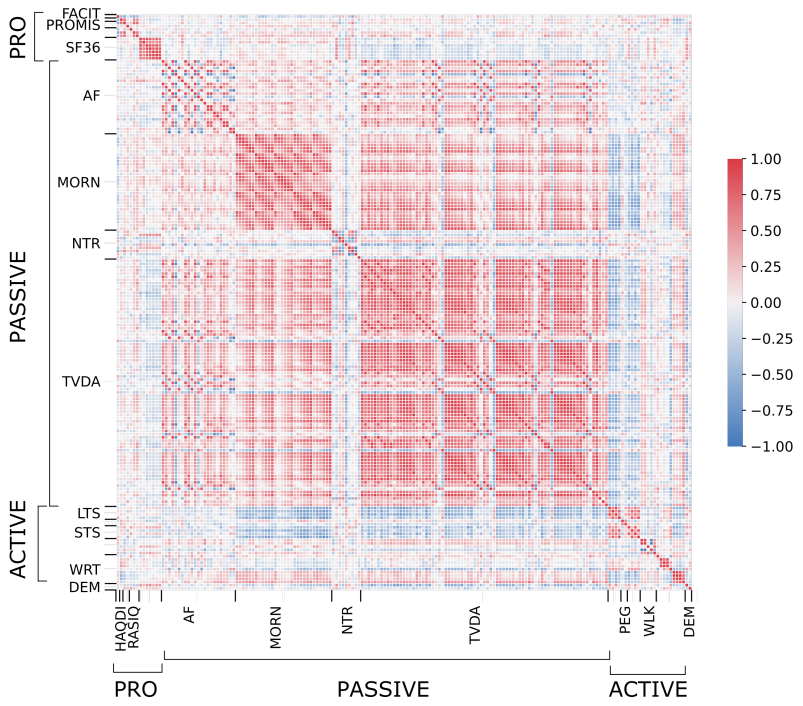

Supplementary Figure 3. Assessing correlation and collinearity between PRO and sensor-based features. Pairwise Spearman's ρ correlation matrix for PRO, active, and passive features, labelled by feature domain. Feature association is bounded between +1/-1 denoting positive and negative correlation. Feature domain abbreviations: FACIT: Functional Assessment of Chronic Illness Therapy Fatigue; HAQ-DI: Health Assessment Questionnaire-Disability Index; PROMIS: Patient-Reported Outcomes Measurement Information System; RASIQ: GSK RA symptom and impact questionnaire; SF-36: Short-Form 36 (SF-36); AF: activity fragmentation; MORN: morning stiffness; NTR: night-time restlessness; TVDA: total volume of daytime activity; LTS: lie-to-stand assessment; PEG: 9-hole peg test; STS: sit-to-stand assessment; WRT: wrist assessment; WLK: walking assessment; DEM: demographics.

also revealed good-to-excellent ($\rho > 0.75$) correlation between domains of features sources (inter-source); for example, TVDA features were not only correlated with each other, but with other passive feature domains, such as AF or MORN features. Much of the inter-source correlation was between similar domains, such as within the activity monitoring-based feature domains, or within the guided test (active) feature domains—suggesting a high degree of multicollinearity and redundancy. However, mostly fair correlations (ρ=0.25–0.50) between active and passively extracted sensor features suggested that different information may be learned during activity monitoring versus guided test exercises. The resulting correlation matrix is depicted in Supplementary Fig. 3.

Assessing feature importance Table 2 represents a selection of features that were retained by LR-elastic-net. The model tended to pick features from all domains, but consistently tended to select many different features between cross validation splits. Some features however, for example, the mean transition time [sec] from standing to lying, daily average total time in MVPA bouts [mins], the average hazard of non-MVPA to MVPA bouts were constantly chosen over all data splits. Other features were selected less often but, when chosen, weighted highly in the model, for instance: median ROM [deg] or the number of movement episodes during night-time sleep [count/hr].

The top selected features from the PRO and sensor-based outcomes fusion model are described in table 3. It was observed that features from all sources: PRO, active, and passive, were prominently selected to estimate RA severity levels. For example, patient-reported RASIQ joint pain; the guided test-based 9HPT total time and ROM velocity; and the volume walking, as measured by the continuous number of walking periods with duration >30 minutes (with up to 1-minute rest period), were always selected to stratify RA severity.

Assessing feature reliability In order to determine the test-retest reliability of the selected features, we calculated intra-class correlation coefficient (ICC) values[1], which were used to assesses the degree of similarity between repeated features over the

Supplementary Table 2. Selection of top performing active (smartphone) and passive (smartwatch) extracted features for RA identification, as determined by logistic regression (LR) elastic-net across 5-fold subject-wise cross validation (CV), with fortnightly (i.e., study duration) averaged features. Features were ranked per CV fold by increasing shrinkage regularisation parameter λ and recording the percentage (%) of time that feature is selected in the subset that minimises the CV error in the validation set. Feature domain abbreviations: AF: activity fragmentation; DEM: demographics; LTS: lie-to-stand assessment; MORN: morning stiffness; NTR: night-time restlessness; STS: sit-to-stand assessment; TVDA: total volume of daytime activity; WLK: walking assessment; WRT: wrist assessment.

	w[1]	p[a]	ICC	MDC$_{95}$%	selected	source	domain	metric
1	0.235±0.140	0.015	0.977 [0.98, 0.99]	13.6%	100%	phone	LTS	Mean transition time from standing to lying [sec].
2	-0.134±0.063	<0.001	0.820 [0.74, 0.88]	30.5%	100%	watch	AF	Average hazard of non-MVPA to MVPA bouts.
3	-0.121±0.095	<0.001	0.818 [0.74, 0.88]	197.6%	100%	watch	TVDA	Daily average total time in MVPA bouts [mins].
4	-0.117±0.058	<0.001	0.862 [0.80, 0.91]	147.7%	100%	watch	TVDA	Median acc. magnitude while in MVPA bouts [mg].
5	-0.096±0.068	<0.001	0.820 [0.74, 0.88]	194.6%	100%	watch	AF	Average consecutive duration in MVPA bouts [mins].
6	0.237±0.180	0.003	0.986 [0.98, 0.99]	13.6%	80%	phone	LTS	Mean transition time from lying to standing [sec].
7	-0.113±0.104	<0.001	0.902 [0.86, 0.94]	55.6%	80%	watch	TVDA	Daily percent of time spent walking.
8	-0.060±0.055	<0.001	0.872 [0.82, 0.92]	143.2%	80%	watch	TVDA	Average acc. while in MVPA [mg].
9	0.187±0.196	0.072	0.982 [0.97, 0.99]	3.0%	60%	phone	WRT	Median range-of-motion (ROM) [deg]
10	0.092±0.116	0.14	0.833 [0.76, 0.89]	69.3%	60%	watch	MORN	SD acc. value 30 mins after wake-up [mg].
11	0.069±0.081	0.109	0.618 [0.46, 0.75]	147.1%	60%	watch	NTR	Midpoint of night-time sleep window [hours]
12	0.184±0.267	0.154	0.735 [0.62, 0.83]	220.2%	40%	watch	NTR	# of movements during night-time sleep [count/hr].
13	-0.083±0.177	0.741	0.795 [0.71, 0.87]	16.5%	40%	watch	NTR	Awake period during night-time sleep [mins].
14	0.079±0.114	0.273	0.987 [0.98, 0.99]	9.8%	40%	phone	STS	Mean transition time from sitting to standing [sec].
15	-0.030±0.059	0.015	0.985 [0.98, 0.99]	17.2%	40%	phone	WRT	Range-of-motion (ROM) median velocity [deg/sec].

Abbreviations (Abbrv.): acc., acceleration; sec, seconds; mins, minutes; h, hours; deg, degrees; deg/sec, degrees per second; ROM, range of motion; m, meters; m/s, meters per second; mg: mili-gravity units of acceleration; SD, standard deviation.
[1] Refers to the mean ± standard deviation in LR feature coefficient values, w, over all CV folds;
[a] Differences in feature distributions between RA / non-RA participants were investigated using a Mann-Whitney U Test.
p-values were post-hoc corrected using methods described by Benjamini and Hochberg[5];
ICC, intraclass correlation coefficient [95% confidence interval];
MDC$_{95\%}$, minimal detectable change (in percent, %).

course of the study for each patient. Here, we calculated $ICC(3,k)$[2]–which considers the two-way random average measures with k repeated measurements—for the 14-day session across subjects, where the raters k are the study days. The minimal detectable change, with a 95% confidence interval (CI) (MDC$_{95}$), was also calculated to determine the minimal change in a feature which is greater than the within subject variability and measurement error, indicating how much a measured change is likely to reflect true change from repeated measurement. First, the standard error of measurement (SEM), which provides an absolute index of precision[3] was calculated, $SEM = SD \times \sqrt{(1-ICC)}$, where SD and ICC are the variance and intra-class correlation coefficients of the feature, x. Next, the minimal detectable change, with a 95% confidence interval (CI) (MDC$_{95}$) was determined[3,4]:

$$MDC_{95} = 1.96 \times SD \times \sqrt{(1-ICC)} \times \sqrt{2} \tag{1}$$

where MDC_{95} was expressed as percentages that are independent of the units of measurement for each feature:

$$MDC_{95}\% = \frac{MDC_{95}}{\bar{x}} \tag{2}$$

using the respective mean feature value, $\bar{x}$. Planned future work will aim to assess the sensitivity of the features extracted to longitudinally & remotely monitor RA.

Evaluation of machine learning estimation of RA status and severity The following figures expand the characterisation of our machine learning estimation of RA status and severity. Table 4 compares model performance distinguishing RA participants from healthy controls, using various data source and feature combinations. Table 5 compares the same task performance using various linear and non-linear model-types, using fortnightly (i.e., study duration) averaged active + passive features. Lastly, table 6 compares the performance of logistic regression models for classification of RA severity levels between different regularisation approaches, for combinations of patient reported outcomes (PROs) augmented with combinations of active and passive sensor-based features.

Supplementary Table 3. Top 10 selected features from PRO + sensor-outcome based RA severity level estimation, as determined by LR-elastic-net across 5-fold subject-wise cross validation (CV), with fortnightly (i.e., study duration) averaged features.

	w[1]	p[a]	selected	source	domain	metric
1	1.876±0.670	0.002	100%	PRO	RASIQ	Joint pain
2	1.696±1.066	0.07	100%	phone	PEG	9HPT total time [sec]
3	1.324±0.762	0.45	100%	-	DEM	Age range [5 years)
4	1.177±1.128	0.13	100%	phone	WRT	Range-of-motion (ROM) median velocity [deg/s]
5	0.971±0.774	0.09	100%	watch	TVDA	# continuous periods of walking > 30 mins [count]
6	-0.410±0.276	0.53	100%	-	DEM	Sex [M/F]
7	-0.965±0.897	0.10	80%	phone	WRT	Range-of-motion (ROM) [deg]
8	0.543±0.825	0.07	80%	PRO	RASIQ	Joint stiffness
9	0.430±0.410	0.12	80%	watch	AF	Average hazard of sedentary to non-sedentary bouts
10	0.904±0.887	0.02	60%	PRO	HAQ	HAQ-DI total score

Abbreviations (Abbrv.): PRO, patient-reported outcome; DEM, demographics information; acc., acceleration; s, seconds; mins, minutes; h, hours; deg, degrees; deg/s, degrees per second; ROM, range of motion; m, meters; m/s, meters per second; mg: mili-gravity units of acceleration.
[1] Refers to the mean ± standard deviation in LR feature coefficient values, *w*, over all CV folds;
[a] Differences in feature distributions between RA (mod) / RA (sev) participants were investigated using a Mann-Whitney U Test. *p*-values were post-hoc corrected using methods described by Benjamini and Hochberg[5];

Supplementary Table 4. Comparison of RA vs. HC classification performance across different source and feature combinations with 5-fold cross-validation (CV). Results are presented as: (1) the posterior overall *subject-wise* outcome for one cross-validation (CV) run as well as (2) the *observation-wise* median and inter-quartile range (IQR) across that CV in brackets. The best performing model for each source combination are highlighted in **bold**. auroc: Area under the receiver operator curve; κ, Cohen's Kappa statistic; F_1, Macro-F1 score.

source	feature[1]	model	auroc	κ	F_1
active	daily	LR-elastic-net	0.673 (0.681, 0.616–0.748)	0.183 (0.180, 0.090–0.421)	0.531 (0.648, 0.464–0.676)
		LR-SG-lasso	0.817 (0.725, 0.676–0.930)	0.542 (0.433, 0.258–0.708)	0.755 (0.680, 0.610–0.831)
	weekly	LR-elastic-net	0.699 (0.757, 0.629–0.792)	0.362 (0.374, 0.118–0.459)	0.640 (0.667, 0.444–0.700)
		**LR-SG-lasso**	**0.802 (0.771, 0.729–0.825)**	**0.614 (0.471, 0.408–0.545)**	**0.800 (0.667, 0.667–0.762)**
	fortnightly	LR-elastic-net	0.779 (0.743, 0.686–0.800)	0.333 (0.267, 0.098–0.633)	0.655 (0.667, 0.545–0.800)
		LR-SG-lasso	0.795 (0.743, 0.714–0.833)	0.578 (0.500, 0.471–0.633)	0.778 (0.727, 0.667–0.800)
passive	daily	LR-elastic-net	0.821 (0.691, 0.680–0.701)	0.439 (0.347, 0.323–0.362)	0.714 (0.689, 0.617–0.691)
		LR-SG-lasso	0.852 (0.712, 0.677–0.758)	0.609 (0.369, 0.335–0.380)	0.776 (0.691, 0.667–0.698)
	weekly	LR-elastic-net	0.837 (0.795, 0.722–0.859)	0.579 (0.441, 0.438–0.507)	0.786 (0.706, 0.667–0.733)
		LR-SG-lasso	0.812 (0.833, 0.804–0.906)	0.612 (0.588, 0.571–0.607)	0.795 (0.750, 0.714–0.828)
	fortnightly	**LR-elastic-net**	**0.790 (0.800, 0.657–0.943)**	**0.615 (0.500, 0.500–0.814)**	**0.807 (0.727, 0.727–0.889)**
		LR-SG-lasso	0.831 (0.867, 0.657–0.943)	0.579 (0.500, 0.314–0.814)	0.786 (0.727, 0.714–0.889)
active + passive	daily	LR-elastic-net	0.814 (0.729, 0.704–0.891)	0.473 (0.312, 0.196–0.552)	0.727 (0.686, 0.611–0.767)
		LR-SG-lasso	0.837 (0.744, 0.704–0.908)	0.504 (0.317, 0.275–0.625)	0.720 (0.683, 0.605–0.808)
	weekly	LR-elastic-net	0.848 (0.842, 0.786–0.850)	0.614 (0.538, 0.486–0.577)	0.800 (0.769, 0.762–0.800)
		LR-SG-lasso	0.848 (0.833, 0.700–0.893)	0.614 (0.589, 0.254–0.814)	0.800 (0.783, 0.692–0.889)
	fortnightly	LR-elastic-net	0.857 (0.914, 0.867–0.914)	0.579 (0.471, 0.351–0.814)	0.786 (0.714, 0.667–0.889)
		**LR-SG-lasso**	**0.842 (0.867, 0.714–0.943)**	**0.615 (0.676, 0.500–0.814)**	**0.807 (0.833, 0.727–0.889)**

[1] daily: daily feature values over the 14-day study period; weekly: the average daily feature value over a 7-day period; fortnightly: the average daily feature value over a 14-day period;

Supplementary Table 5. Comparison of RA vs. HC classification performance for logistic regression (LR) based models and decision trees (DT) across with 5-fold cross-validation (CV) with fortnightly (i.e., study duration) averaged active + passive features. Results are presented as: (1) the posterior overall *subject-wise* outcome for one cross-validation (CV) run as well as (2) the *observation-wise* median and inter-quartile range (IQR) across that CV in brackets. The best performing model for each feature representation are highlighted in **bold**. auroc: Area under the receiver operator curve; κ, Cohen's Kappa statistic; F_1, Macro-F1 score.

	model	auroc	κ	f1
LR	-	0.853 (0.867, 0.800–0.943)	0.542 (0.500, 0.459–0.814)	0.755 (0.727, 0.727–0.889)
	lasso	0.788 (0.857, 0.667–0.867)	0.545 (0.500, 0.241–0.814)	0.772 (0.727, 0.714–0.889)
	ridge	0.777 (0.833, 0.767–0.914)	0.612 (0.657, 0.441–0.814)	0.792 (0.800, 0.769–0.889)
	elastic-net	0.801 (0.867, 0.667–0.914)	0.612 (0.657, 0.441–0.814)	0.792 (0.800, 0.769–0.889)
	**SG-lasso**	**0.842 (0.867, 0.714–0.943)**	**0.615 (0.676, 0.500–0.814)**	**0.807 (0.833, 0.727–0.889)**
DT	RF	0.862 (0.933, 0.829–0.957)	0.646 (0.657, 0.471–0.814)	0.800 (0.800, 0.727–0.889)
	XGB	0.851 (0.833, 0.829–0.914)	0.614 (0.676, 0.471–0.814)	0.800 (0.833, 0.714–0.889)

Supplementary Table 6. Comparison of RA severity level prediction using patient reported outcomes (PRO), versus using PRO + sensor-outcomes, over 5-fold cross-validation (CV) with fortnightly (i.e., study duration) averaged active + passive features. Results are presented as: (1) the posterior overall *subject-wise* outcome for one cross-validation (CV) run as well as (2) the *observation-wise* median and inter-quartile range (IQR) across that CV in brackets. The best performing model for each feature representation are highlighted in **bold**. auroc: Area under the receiver operator curve; κ, Cohen's Kappa statistic; F_1, Macro-F1 score.

features	model	auroc	κ	f1
PRO	LR-SG-lasso	0.736 (1.000, 0.750–1.000)	0.403 (0.286, 0.286–0.667)	0.733 (0.667, 0.667–0.857)
	LR-elastic-net	0.819 (1.000, 0.750–1.000)	0.479 (0.286, 0.286–0.615)	0.759 (0.750, 0.667–0.800)
PRO + active	LR-SG-lasso	0.747 (1.000, 0.833–1.000)	0.327 (0.286, 0.118–0.615)	0.710 (0.667, 0.667–0.800)
	LR-elastic-net	0.901 (1.000, 0.833–1.000)	0.560 (0.545, 0.333–1.000)	0.750 (0.667, 0.571–1.000)
PRO + passive	LR-SG-lasso	0.791 (1.000, 0.900–1.000)	0.479 (0.286, 0.286–1.000)	0.759 (0.667, 0.667–1.000)
	LR-elastic-net	0.879 (1.000, 0.925–1.000)	0.555 (0.545, 0.286–0.667)	0.786 (0.800, 0.750–0.857)
PRO + active + passive	LR-SG-lasso	0.885 (1.000, 0.906–1.000)	0.479 (0.286, 0.286–0.545)	0.759 (0.667, 0.667–0.750)
	**LR-elastic-net**	**0.907 (1.000, 0.889–1.000)**	**0.707 (1.000, 0.667–1.000)**	**0.833 (1.000, 0.667–1.000)**

Supplementary Methods

Remotely collected smartphone and smartwatch sensor data was obtained from the GSK study title: Novel Digital Technologies for the Assessment of Objective Measures and Patient Reported Outcomes in Rheumatoid Arthritis Patients: A Pilot Study Using a Wrist-Worn Device and Bespoke Mobile App. (212295, weaRAble-PRO)[6]. This observational study followed 30 participants diagnosed with moderate-to-severe RA and 30 matched HCs over 14 days. Supplementary Figure 4 details the frequency and timeline of data capture for each data source in the weaRAble-PRO Study.

Inclusion and exclusion criteria For participation in the full pilot study, 30 RA participants and 30 HCs matched on age, gender, and race were recruited. Due to the small sample size and potentially limited pool for recruitment, ages were matched within a window of $\pm$ 3 years. The overall ratio of moderate to severe participants was chosen not to exceed 2:1 in either direction. Inclusion/Exclusion criteria were the same for RA participants and HCs, unless otherwise noted. All participants must have been able and willing to perform the pre-defined guided tests at the start of the study, follow audio instructions from an iPhone, and have a sufficient level of English language to ensure ability to understand mobile app and questionnaires. Rheumatoid Arthritis participants were at least 21 years of age at date of consent for study, were selected based on clinically verified diagnosis of moderate-to-severe RA, with severity assessed using Routine Assessment of Patient Index Data 3 (RAPID3). Healthy controls were selected based on no prior or current diagnosis of a rheumatological disorder, inflammatory disorder, malignancy, or other relevant diseases. Further exclusion criteria for all participants included history of other inflammatory rheumatologic or systemic autoimmune disorder (e.g., Hashimoto's thyroiditis or Sjogren's syndrome), history of movement disorders, other neurological disorders or conditions resulting in significant physical impairments that impact joint movements to be assessed, history of postural hypotension, unexplained syncope, or other conditions that make it difficult for participants to perform guided tests such as the lie-to-stand test and any history of severe skin allergy. Participants were also excluded if they required use of a wheelchair, walking aids, artificial limbs, or had any active implantable device, such as a pacemaker.

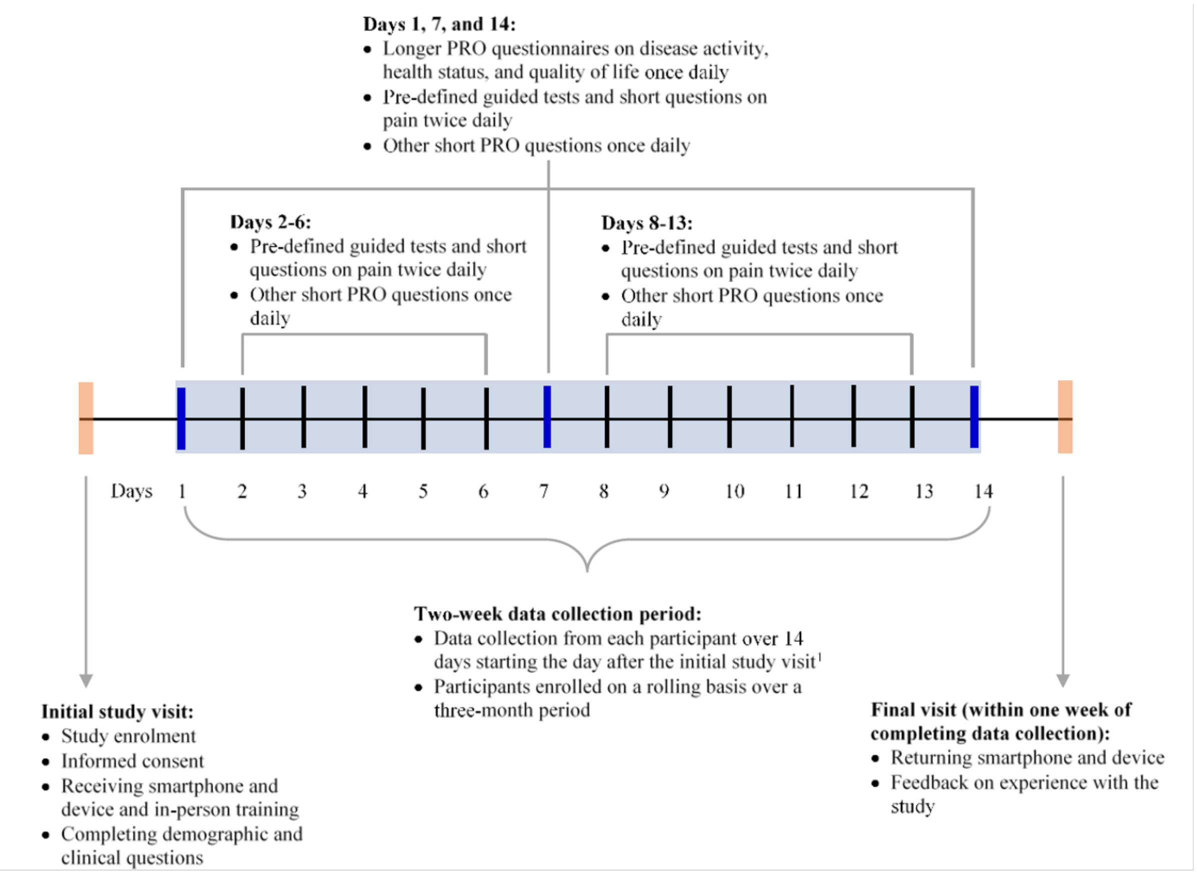

Supplementary Figure 4. Overview of frequency and timeline of data captured.

Patient-reported outcomes Patient-reported outcomes (PRO), most often self-report questionnaires, were administered to assess disease activity, symptoms, and health status and quality of life from the patients' perspective[7,8]. A selection of validated PRO measures for RA were administered on days 1, 7, and 14 of data collection, as described in table 7.

Supplementary Table 7. Overview of the PRO assessments administered to RA and HC participants on days 1, 7, and 14 in the weaRAble-PRO study

PRO	Abbrv.	Domain(s) assessed
GSK RA symptom and impact questionnaire	RASIQ	Generalised measure of the severity of RA symptoms and their impact on the patient[9].
Patient-Reported Outcomes Measurement Information Systemt[1]	PROMIS pain<br><br>PROMIS sleep	Pain interference developed to assess the degree to which pain interferes with participants' physical, mental, and social activities[10].<br>Sleep disturbance as assessed self reported perceptions of sleep quality, including perceived difficulty in falling asleep, difficulty experienced in staying asleep, sleep depth, and satisfaction with sleep quality[11].
Health Assessment Questionnaire-Disability Index	HAQ-DI	Self reported functional status measures. It is one of the most widely used measure of function in RA, with demonstrated reliability and validity in RA patients[12].
Functional Assessment of Chronic Illness Therapy Fatigue	FACIT	Assesses four domains of fatigue[13]: physical fatigue, functional fatigue, emotional fatigue, social consequences. This questionnaire has been validated for use with RA patients[14,15].
Short-Form 36	SF-36	36-item questionnaire to allow participants to self-assess functional health and well-being[16]. Scores are provided for eight domains: general health, mental health, physical functioning, social functioning, physical role, emotional role, bodily pain, vitality. This instrument has been validated and used in studies with RA patients[17,18].
Interactive joint-pain map[2]	JMAP	Records the number and severity of up to 55 pre-specified joints experienced by the participant at a given time. For the joints where patients are experiencing any pain at the given moment, they are asked to score the level of pain as 1 (mild pain), 2 (moderate pain), or 3 (severe pain)[19].
Visual analogue scale[2]	VAS	Patient's assessment of arthritis pain is a single-item question that assesses the level of pain severity the participant is currently experiencing using a visual analogue scale ranging from 0 to 100[20].

Abbrv., abbreviations
[1] An reduced set of items from the PROMIS item bank for pain and sleep domains were used for this study;
[2] Administered daily over the 14-day study, within an hour of completing the predefined guided tests, once in the morning, and once in the afternoon.

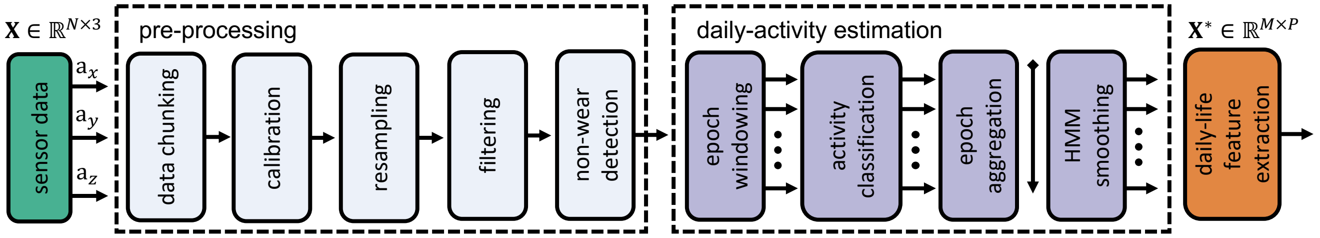

Supplementary Figure 5. Sensor processing pipeline developed for the Apple Watch in the weaRAble-PRO study. The raw 3-axis accelerometer sensor data, $(\mathbf{a}_x, \mathbf{a}_y, \mathbf{a}_z)$, denoted by $\mathbf{X} \in \mathbb{R}^{N\times3}$, where N are the number of continuously collected accelerometer samples over the 14-day study period ($N = 50\ [Hz] \times 3600\ [sec] \times 24\ [hr] \times 14\ [days]$), can be transformed into measures characterising physical activity and sleep, $\mathbf{X}^* \in \mathbb{R}^{N\times3}$, where M is the new sampling range, *daily* ($M = 14$), and P are the number of measures of daily life (i.e., features). In this case, a $N \gg M$ problem has been reduced into useful $M \times P$ features, unobtrusively measuring the physical activity and sleep of RA participants during daily life.

Smartwatch sensor feature extraction

The sensor processing pipeline developed for the Apple Watch in the weaRAble-PRO study is outlined in Supplementary Fig. 5, yielding unobtrusively measured summary features of physical activity and sleep for RA participants, computed daily during normal life.

An overview of the pipeline is as follows:

1. **Pre-processing:**
   (a) Data chunking, memory optimisation. Convert raw 50 Hz accelerometer data to daily chunks;
   (b) Calibration to local gravity, local UTC timestamps;
   (c) Resampling, 30 Hz;
   (d) Butterworth, low-pass filtering at 17 Hz;
   (e) Non-wear detection and segmentation;
2. **Daily-activity Estimation:**
   (a) Epoch windowing, 30 [sec];
   (b) Activity classification per epoch;
   (c) Epoch aggregation, daily;
   (d) Posterior activity prediction with hidden Markov model (HMM) smoothing;
3. **Characterising daily life:**
   (a) Physical activity and sleep feature extraction, see section for more details;

Deep Network-based Activity Recognition In this work, a deep learning-based activity recognition model, known as a deep convolutional neural network (DCNN), was trained on Capture-24 and then used to directly estimate daily activity in the weaRAble-PRO study.

Multi-Task Self-Supervised Learning Developing robust activity classification models is challenging in clinical studies due to the lack of labelled data for training. Deep networks, in particular, need a lot of training data in order to be robust and generaliseable. Open-source HAR-based datasets have small sample sizes, with generally n<100 participants as annotating free-living wearable data for human activity recognition (HAR) requires a concurrent video stream, and the labelling process is resource-intensive[21]. There are however massive-scale unlabelled wearable datasets, such as the UK Biobank (UKB), which have collected data on roughly 100,000 participants with over 6 billion samples available. This study build upon our previous work demonstrating how advances in self-supervised learning (SSL) could help exploit the hidden information in these large-scale unlabelled datasets[21]. SSL consists of training a model on a pretext task in an unlabelled dataset (often in a multi-task problem). The supervised task is devised based on labels manually created in the unlabelled dataset, such as distinguishing transformed versus original data. The SSL model has to determine for each sample, if a transform has been applied or not, and what transform(s) have been applied as a multi-task problem. Essentially you create a robust deep feature

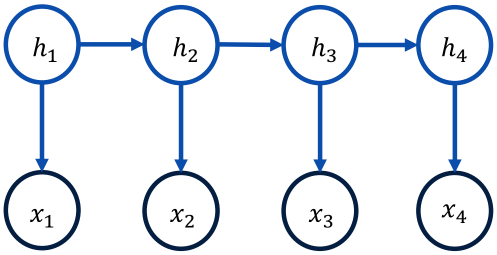

Supplementary Figure 6. Diagram of a Hidden Markov Model (HMM). The sequence of discrete hidden states $\mathbf{h} = \{h_1, \ldots, h_{(t-1)}, h_t, h_{(t+1)}, \ldots h_N\}$ form a Markov chain. At each time step an observation is obtained by a draw from a probability distribution that is conditional on the value of h at that time. This results in a sequence of observable values $\mathbf{x} = \{x_1, \ldots, x_{(t-1)}, x_t, x_{(t+1)}, \ldots, x_N\}$.

extractor, built on a diverse and large amount of data. This pre-trained model can then be fine-tuned on a downstream task, such as activity recognition in the smaller datasets, such as Capture-24. In the main text, Fig. 7 illustrates a multi-task self-supervised approach for feature learning in HAR. We treated each of the tasks as a binary problem predicting whether a self-supervised transformation has been applied. Our multi-task SSL training relied on the *unlabelled UKB*, which contains roughly 700,000 person-days of free-living activity data (100,000 participants, 7 days of wear). For more information we refer the reader to our previous work[21].

Deep Network Architecture We used a deep convolutional neural network (DCNN) with a ResNet-V2 architecture, consisting of 18 layers and 1D convolutions[22] as a feature extractor (10M parameters). The learned feature vector was of size 1024. All the tasks will share the same feature extractor. Then, we attached a softmax layer for each of the self-supervised tasks. In the downstream evaluation, we added a fully-connected (FC) layer of size 512 in between the feature extractor and softmax readout. The network structure was fixed for all the downstream evaluations. We computed the cross-entropy loss for each task and weighed all the tasks equally in loss calculation.

Hidden Markov Model (HMM) smoothing Human activity recognition (HAR) model predictions are essentially independent—meaning that the sequence of activities over each 30 second epoch incorporates no temporal information epoch-to-epoch, for instance how the previous epoch prediction affects the current, or next, activity prediction. In order to add temporal dependency to the human activity recognition (HAR) model developed a Hidden Markov Model (HMM) was implemented in a post-processing step to obtain a more accurate sequence of predicted activities over the continuous 14-day data collection period. The HMM defines a Markov chain on hidden (or "latent") variables $h_t = \{h_1, h_2, ..., h_H\}$, such that only the recent past influences the future:

$$p(h_t | h_{(1:t-1)}, ..., h_{(t-1)}) = p(h_t | h_{t-1})$$

The observed (or "visible") variables are dependent on the hidden variables through an emission $p(x_t|h_t)$. This defines a joint distribution:

$$p(\mathbf{h}|\mathbf{x}) = p(x_1|h_1)p(h_1) \prod_{t=2}^{N} p(x_t|h_t)p(h_t|h_{t-1}) \tag{3}$$

where $p(x_t|h_t)$ defined the emission probability; $p(h_t|h_{t-1})$ defines the transition probability between hidden states; The transition distribution $p(h_{t+1}|h_t)$ is defined by a $H \times H$ transition matrix: $\mathbf{A}_{i',i} = p(h_{t+1} = i'|h_t = i)$. The emission distribution, $p(x_t|h_t)$, has discrete states $x_t \in \{1, ..., V\}$, we can define a $V \times H$ emission matrix: $\mathbf{B}_{i,j} = p(x_t = i|h_t = k)$. For continuous outputs, h_t selects one of H possible output distributions $p(x_t|h_t)$, $h_t \in \{1, ..., H\}$. The most likely hidden path, i.e., sequence of states, $\arg\max_{\mathbf{w}}\{p(\mathbf{h}|\mathbf{x})\}$, is then found via the is found via the Viterbi algorithm. A diagram of a HMM is shown in Supplementary Fig. 6. At each time step t, we define h_t as one of k activity classes, $\{c_1, c_2, ..., c_k\}$ as {sleep, sedentary, light physical activity, moderate-to-vigorous physical activity (MVPA)}. While h_t are not observed directly, at each t step there is an dependent observed stochastic emission x_t. The hidden state sequence $\mathbf{h}$ is defined as the true activity labels and the emission distribution $p(x_t|h_t)$ is estimated directly using the predicted activity probabilities from the HAR model in the training set. As such we use the training predictions of activity from HAR model to infer the most likely sequence of true activity states that would have given rise to those predictions. HMM smoothing helps to correct for erroneous predictions, such as when the transitions between those two classes of activity are rare, for instance sleeping to walking.

Passive Features Here we detail the passively extracted, activity monitoring-based features in the weaRAble-PRO study. Activity monitoring features were developed based on broad activity prediction labels {sleep, sedentary, light physical activity, moderate-to-vigorous physical activity (MVPA)}[23,24] and fine-grained activity prediction labels {sleep, sitting/standing, mixed, vehicle, walking, bicycling}[25]. Measures of physical activity and sleep were summarised based on smartwatch actigraphy sensor data magnitude data per epoch, or aggregated by intensity levels, activity classification and bouts of activity. These features could be broadly grouped into physical activity domains: total volume of daytime activity (TVDA), fragmentation of activity (AF), along with two RA symptom-specific domains focusing on morning stiffness (MORN) and night-time restlessness (NTR):

1. **Total volume of daytime activity (TVDA)**: captures information around the overall physical activity and the ability to perform physical activity at varying levels of intensity during daytime, which are known to be altered in patients with RA[26,27];

2. **Activity Fragmentation (AF)**: Metrics in this domain attempt to capture information related to the ability to perform sustained activity. Frequent interruptions of physical activity may reflect a worse health condition. For example, RA patients may need to interrupt some activity due to increased joint pain.

3. **Morning stiffness (MORN)** is a common symptom of RA. These measures include information related to timing of activity after getting up in the morning as estimated from the activity classification[28].

4. **Night-time Restlessness (NTR)**: These measures include information related to timing, duration and quality of sleep. There is evidence that RA patients experience fluctuations in disease activity following a circadian rhythm with worsening of the illness during the night[29], thus measures of overnight movement serve as a proxy for estimating night-time restlessness, reflecting the impact of the disease on sleep quality.

Smartphone sensor feature extraction

Peg Test Algorithm The 9-hole peg test is a two-step test of hand dexterity to measure the MSFC score in Multiple Sclerosis, or signs of Parkinson's disease or stroke[30]. The digital 9-hole peg test (9HPT), and subsequent metrics calculated, are proprietary to Apple ResearchKit, see http://researchkit.org/docs/docs/ActiveTasks/ActiveTasks.html for more details. This smartphone version of the 9HPT task requires participants to use two fingers to touch the on-screen peg and drag it into the an on-screen hole. Once all 9 pegs have been placed in each hole, the task is repeated by removing the pegs in the same manner. The 9HPT is repeated using both dominant and non-dominant hands. The total duration that the user spent taking the test for each hand repetition is recorded.

Wrist ROM Test Algorithm The wrist range of motion (ROM) test algorithm is outlined previously as part of the GSK PARADE study[19,31]. The iPhone accelerometer sensor data is converted to angular positions; ROM is then computed based on the differences between angular maxima; angular velocity is determined from the gyroscope sensors.

Walk Test Algorithm The gait test algorithm is outlined previously as part of the GSK PARADE study[19]. During walking, initial (IC) and final (FC) feet contact timings are calculated from the smartphone accelerometer data with an inverted pendulum model, as described in[32,33]. Contact points are determined by integration of the vertical component of the accelerometer signal, $\mathbf{a}_y$, and subsequent differentiation of that signal with a continuous wavelet transform (CWT, convolution of the accelerometer data and an analysing function, i.e., mother wavelet). ICs and FCs are then denoted by minima and maxima timings respectively in this transformed signal. Detected peaks were used to estimate the number of steps, cadence, step length[34], and walk velocity for each assessment.

Sit-to-stand Test Algorithm Both the iPhone accelerometer and gyroscope data were used to determined when participants were in a sitting or standing position during the sit-to-stand (STS) exercise[35]. The vertical accelerometer component, $\mathbf{a}_y$, determined sit-to-stand transitions by estimating the phone axis orientation (and change thereof) relative to gravity, as the participants moved between sitting and standing—given the phone's axis should be fixed as it is strapped to the participants thigh using a strap. Gyroscope axis sensor components, $(\mathbf{g}_x, \mathbf{g}_y, \mathbf{g}_z)$, helped determine whether a participant had completed valid transitions during the exercise. The start and end points of the STS transitions could therefore be determined by identifying peaks in $\mathbf{a}_y$ that were within the given thresholds where participants could be feasibly standing or lying. The length of a sit-to-stand transition was then calculated as the difference between the point where the participant first reached a standing position and the final point where the participant was sitting prior to beginning a standing motion.

Supplementary Table 8. Overview of the daily prescribed smartphone-recorded Active Assessments (denoted "Guided Tests") in the weaRAble-PRO study.

Exercise	Abbrv.	Brief Description
Wrist ROM	WRT	Participants were requested to sit down and place their forearm at the edge of a table, holding the iPhone horizontally facing up in their hand, and to flex and extend their wrist joint to its maximum angle, repeating the motion for 10 seconds. The test was carried out twice, once using each hand[19].
Gait	WLK	Participants were asked to affix the iPhone to their leg (on the right thigh) facing outward using a provided strap, then to walk in a straight line for 30 seconds (while being allowed to turn around at any point in the middle of the test)[19].
9-hole peg test	PEG	Digital touch-screen version of the standard clinical assessment where participants are asked to place, and subsequently remove, 9 pegs into and from a round hole, in the fastest time possible[37].
Sit-to-stand	STS	Participants were requested to perform a sit-to-stand transition from a chair with the iPhone attached to their upper right thigh with a strap, repeating the exercise 5 times at their own pace[35,38].
Lie-to-stand	LTS	Participants performed a lie-to-stand transition: from lying still with legs stretched on a bed, to standing up on the floor. Participants were requested to affix the iPhone to their right thigh with a strap during the exercise and repeat twice at their own pace[35,38].

Abbreviations (abbrv.): ROM, range of motion;

Lie-to-Stand Test Algorithm Following a similar STS algorithm[35], during the lie-to-stand test, accelerometer and gyroscope (y-axis) measurements were used to determine a participant's standing and lying transition points following the algorithm introduced in[36]. Gyroscopic y-axis gravity relates the phone's orientation relative to gravity at a given moment in time and the phone acceleration helped to improve accuracy in determining participants' lying positions—for example, the point of minimum acceleration generally corresponded to moments when participants held a lying position (causing a short plateau in y-gravity).

Machine-learning estimation of RA status and severity

Feature Pre-processing Missing data extracted from the GTs and passive monitoring was imputed using a carry-last value forward per participant[39]. In cases where the last value was missing, mean imputation was used instead. Features were assessed for non-normality by visual inspection. Those non-normal features were transformed using box-cox transformations[40]. Features were normalised using the z-score to have unit variance using their respective mean μ and standard deviation $\sqrt{\sigma}$.

Linear Regression A regression model explicitly describes a relationship between predictor(s) $\mathbf{X} \in \mathbb{R}^{N\times P}$ and continuous response variables $\mathbf{y} \in \mathbb{R}^{N}$, the most basic of which is *linear regression* (LR)[41]. For an ith observation row of $\mathbf{X}$, $\mathbf{x} \equiv \mathbf{x_i} \in \mathbb{R}^{1\times P}$:

$$\hat{y} = w_0 + \sum_{j=1}^{P} w_j x_j + \varepsilon \tag{4}$$

$$= \mathbf{w}^\top \mathbf{x} + b \tag{5}$$

where w_j values denote the slope (weights, or regression coefficients) of the x_j features; w_0 is the intercept term; and ε denote the residual (model) errors term, which are assumed to be normally distributed with constant variance, $\varepsilon \sim \mathcal{N}(0,\sigma^2)$[41,42]. Often a linear model is described in vector notation (5), $\mathbf{w} = [w_0, w_1, w_2, \ldots, w_P]$, where w_0 is denoted as the "bias", b term, and the ε-term is often omitted.

Logistic Regression Generalised linear models (GLMs) are extensions of linear regression models that can have non-linear outputs[43]. GLMs utilise canonical link functions, ϕ, to transform the outputs of a linear regression: $\varphi = \mathbf{w}^\top\mathbf{x}$ to another distribution, such as with a logistic $\phi = \sigma(\varphi)$ link function (or inversely the logit, representing the log-odds) which will be used to form Logistic Regression for binary classification tasks[42,44], in this case ϕ is sigmoidal and is bounded between $[0,1]$, therefore the output of σ can be interpreted as the probability of $y = 1$:

$$p(\mathbf{x};\mathbf{w}) = \frac{1}{1+e^{-(\mathbf{w}^\top\mathbf{x})}} \tag{6}$$

A threshold can be applied to the probabilistic output p to determine a classification prediction $\hat{y}$ for a Logistic Regression model; threshold values are typically chosen as 0.5, but this can be altered based on the use case.

Regularisation as Feature Selection Many statistical and machine learning models can easily overfit to the training data, resulting in poorer estimations, models that are not generalisable or too complex. This is likely in the weaRAble-PRO dataset where we have $P \gg N$ problem: the number of predictors P is much larger than the number of observations N (participants), a case where standard models fail. Regularisation can be introduced to mitigate against the $p \gg n$ problem. For example large coefficient values in a regression can be penalised by adding a regularisation term to a loss function, or through reducing the number of parameters or features used in a model. The most common regularisers use the ℓ_p-norm defined by[42,45]:

$$||\mathbf{x}||_p = \left(\sum_{i=1}^{N} |x_i|^p \right)^{1/p} \tag{7}$$

for any $\mathbf{x} \in \mathbb{R}^{N \times 1}$, where the real number $p \geq 1$ defines the ℓ_p space. In this work, we experimented with a number of regularisation techniques in order to perform the classification tasks.

Least Absolute Shrinkage and Selection Operator (LASSO) The Least Absolute Shrinkage and Selection Operator (LASSO)[45,46] is a technique that conversely solves the ℓ_1-penalised sum of squares (7) in a linear regression such that:

$$\hat{\boldsymbol{w}} = \arg\min_{\mathbf{w}} \left\{ ||y - \mathbf{w}^\top \mathbf{x}||_2^2 + \lambda ||\mathbf{w}||_1 \right\} \tag{8}$$

This is equivalent to minimising the sum of squares with a constraint of the form: $||w||_1 = \sum_j^N |w_j| \leq t$. Because of the form of the ℓ_1-penalty, LASSO both shrinks coefficients but also encourages sparsity in a model's parameters and thus inherently forms feature selection, shrinking non-important features to zero. The LASSO can also be extended to perform feature selection for classification by substituting a canonical link function (such as the logistic $\phi = \sigma(\varphi)$) and following the same procedure outlined in equation 6, essentially performing regularised-logistic regression (denoted LR-lasso in this work)[45].

Ridge Regression Ridge Regression (Tikhonov Regularisaion)[42,45] is a technique which utilises the ℓ_2-norm to impose a penalty on the size of the coefficients in a linear regression (7) such that:

$$\hat{\boldsymbol{w}} = \arg\min_{\mathbf{w}} \left\{ ||y - \mathbf{w}^\top \mathbf{x}||_2^2 + \lambda ||\mathbf{w}||_2 \right\} \tag{9}$$

This is equivalent to minimising the sum of squares with a constraint of the form: $||w||_2 = \sum_j^N w_j^2 \leq t$ (where t controls the amounts of shrinkage; there is an exact relationship between t and corresponding λ (denoted LR-ridge in this work).

Elastic Net Elastic net linearly combines the ℓ_1 and ℓ_2 penalties of the lasso and ridge methods such that:

$$\hat{\boldsymbol{w}} = \arg\min_{\mathbf{w}} \left\{ ||y - \mathbf{w}^\top \mathbf{x}||_2^2 + (1-\alpha)\lambda ||\mathbf{w}||_1 + \alpha\lambda ||\mathbf{w}||_2 \right\} \tag{10}$$

given non-negative values λ, and α that is strictly between 0 and 1, which determines the trade off between ℓ_1 and ℓ_2 regularisation (denoted LR-elastic-net in this work).

Sparse-Group LASSO The sparse-group lasso is an extension of the lasso that promotes both group sparsity and within group parameter-wise sparsity, through a group lasso penalty and the lasso penalty:

$$\hat{\boldsymbol{w}} = \arg\min_{\mathbf{w}} \left\{ ||y - \mathbf{w}^\top \mathbf{x}||_2^2 + \lambda_1 ||\mathbf{w}||_1 + \lambda_2 \sum_{l=1}^{M} \sqrt{p_l} ||\mathbf{w}^{(l)}||_2 \right\} \tag{11}$$

where λ_1 is the parameter-wise regularisation penalty and λ_2 is the group-wise regularisation penalty. The data $\mathbf{x}$ contains sub-grouping, such that $\mathbf{x}^{(l)}$, denoting the features in group l, and corresponding learned weights $\mathbf{w}$ containing contains sub-grouping $\mathbf{w}^{(l)}$; p_l is the length of $\mathbf{w}^{(l)}$ (i.e., the number of features in each group) and M are the total number groups. Therefore, the sparse group lasso penalty will yield a sparse set of groups and also a sparse set of covariates in each selected group. As denoted in[47], we use the term "groupwise sparsity" to refer to the number of groups with at least one nonzero coefficient, and "within group sparsity" to refer to the number of nonzero coefficients within each nonzero group (denoted LR-SG-lasso in this work).

Random Forest Classification and Regression Trees (CART), specifically Random Forests (RF), are a multi-functional, non-linear method capable of performing regression, classification and feature selection[48]. Unlike the linear filter-based methods of feature selection, for example, lasso, RFs incorporate non-linear feature selection as part of the model methodology. Random Forests consist of a large ensemble of decision trees arranged in a hierarchical structure. To build an individual tree, we recursively descend through the hierarchy, performing binary splits (decisions) at each level in the structure (a node, j) using a single feature $x_j \in \mathcal{X}^p$ based on a threshold value (splitting criterion) s_j, sub-partitioning the feature space $\mathcal{X}_j$ at each node. A tree is typically expanded until all leaves are pure (i.e each partition $\mathcal{X}_j$ represents only one class) or until all leaves contain less than the minimum number of samples in a partition $\mathcal{X}_j$ required to split a node. An individual tree selects $m < N$ random subset of observations (with replacement), and each node considers a random subset of p features for each split. Once all trees have been grown, each of the 'weaker' decisions are aggregated (or *ensembled*) creating a robust final prediction. For example, consider an ensemble of two trees. The prediction scores of each individual tree are summed to obtain the final prediction:

$$\hat{y}_i = \sum_{k=1}^{K} f_k(x_i), f_k \in \mathcal{F} \tag{12}$$

where K is the number of trees, f_k is a function in the functional space $\mathcal{F}$, and $\mathcal{F}$ is the set of all possible CARTs. The objective function optimised is then given by:

$$E(\theta) = \sum_{i}^{n} \mathcal{L}(y_i, \hat{y}_i) + \sum_{k=1}^{K} w(f_k) \tag{13}$$

where $w(f_k)$ is the complexity of the tree f_k. Classification predictions are deduced as the majority class label of the observations present in each final partition $\mathcal{X}_j$, whereas for continuous prediction (i.e., regression) the mean of the (continuous) responses would be calculated instead. To determine the optimal split criterion s_j for each x_j to create each $\mathcal{X}_j$ we evaluate the *Gini* importance, which quantifies the average gain of purity (i.e., the presence of one class) caused by splits of a given variable. For regression the mean decrease in mean square error (MSE) is assessed instead.

Extreme Gradient Boosted Trees (XGB) Extreme Gradient Boosted Trees (XGB) iterates on the CART through a regularising gradient boosting framework—the difference being in how trees are built and combined. Rather than bagging, like in CART, specifically RF, gradient boosting improves a single weak model by combining it with a number of other weak models in order to generate a collectively stronger model[49]. This boosting is formed as additive strategy during training where a gradient descent algorithm is used to minimise (or maximise) an objective function $E(\theta)$ for each new tree that is added at each time step, t:

$$E(\theta)^t = \sum_{i=1}^{n} \mathcal{L}(y_i, \hat{y}_i^{(t)}) + \sum_{i=1}^{t} w(f_i) \tag{14}$$

where $\hat{y}_i^t$ is the prediction value at step t; and f_i are the parameters of a tree, i.e., the tree structure and the leaf scores that are needed to be learned; and $w(f_i)$ is the complexity of the tree. Therefore $\hat{y}_i^t$ is determined by the prediction of the previous tree at $t-1$, $\hat{y}_i^{(t-1)}$:

$$\hat{y}_i^{(t)} = \sum_{k=1}^{t} f_k(x_i) = \hat{y}_i^{(t-1)} + f_t(x_i) \tag{15}$$

To optimise the error function, XGBoost computes gradients (Jacobian) and Hessians of the error, denoted below as:

$$g_i = \partial_{\hat{y}_i^{(t-1)}} \mathcal{L}(y_i, \hat{y}_i^{(t-1)}) \tag{16}$$

$$h_i = \partial^2_{\hat{y}_i^{(t-1)}} \mathcal{L}(y_i, \hat{y}_i^{(t-1)}) \tag{17}$$

which can be subbed into the objective function in equation (14), yielding:

$$E(\theta)^t = \sum_{i=1}^{n} \left[g_i f_t(x_i) + \frac{1}{2} h_i f_i^2(x_i) \right] + w(f_t) \tag{18}$$

Next, to define the complexity of the tree $w(f)$, we first refine the function for the definition of a tree $f(x)$ as:

$$f_t(x) = w_{q(x)}, w \in \mathcal{R}^T, q : \mathcal{R}^d \rightarrow \{1, 2, ..., T\} \tag{19}$$

where w is the vector of scores on leaves, q is a function assigning each data point to the corresponding leaf, and T is the number of leaves. Then, in XGBoost, we can define the complexity over the number of leaves, T:

$$w(f) = \gamma T + \frac{1}{2}\lambda \sum_{j=1}^{T} w_j^2 \tag{20}$$

where γ and λ are tune-able regularisation parameters. Subbing equation 20 into equation 18, becomes:

$$E(\theta)^t = \sum_{j=1}^{T} \left[G_j w_j + \frac{1}{2}(H_j + \lambda) w_j^2 \right] + \gamma T \tag{21}$$

where $I_j = \{i|q(x_i) = j\}$; $G_j = \sum_{i \in I_j} g_i$; $H_j = \sum_{i \in I_j} h_i$. Following the convention in Chen et al. 2016[49], equation 21 can be reformulated as solving over:

$$w_j^* = -\frac{G_j}{H_j + \lambda} \tag{22}$$

$$E(\theta)^* = -\frac{1}{2} \sum_{j=1}^{T} \frac{G_j^2}{H_j + \lambda} + \gamma T \tag{23}$$

Summarising, XGB iteratively trains an ensemble of shallow decision trees, with each iteration using the error residuals of the previous model to fit the next model. The objective $E(\theta)^*$ measures how good a tree structure $q(x)$ is, optimising one level of the tree at a time, while regularising on the model complexity. For example, to split a leaf into two leaves, and the score will be:

$$Gain = \frac{1}{2} \left[\frac{G_L^2}{H_L + \lambda} + \frac{G_R^2}{H_R + \lambda} - \frac{(G_L + G_R)^2}{H_L + H_R + \lambda} \right] - \lambda \tag{24}$$

if the gain acquired by adding a branch is smaller than γ, it would not be added.

Parameter Tuning Optimal model parameters were determined via randomised grid-search over internal 5-fold (subject-wise) CV with 500 iterations. In the case of XGB, early stopping was determined using roughly 10% of the training data, proportionally, as validation, with for 10 boosting rounds. For regularised logistic regression models, a parameter search was determined over ℓ_1 and ℓ_2 regularisation terms on weights, $\lambda \in \{10^{-5}, ..., 10^{-1}, ..., 0, ..., 10^0, 10^1, ..., 10^5\}$; and the elastic-net mixing parameter $\alpha \in \{0, 0.1, ..., 1\}$; RFs have relatively little hyperparameter tuning: the number of trees to build $p \in \{500, 1000, 1500\}$; the number of input variables chosen at each node $p \in \{\sqrt{P}, 2\sqrt{P}, \sqrt{P}/2\}$, where P are the number of features, as suggested in[50,51]. For XGB, a parameter search was determined over: the boosting learning rate $p \in \{0.01, 0.05, 0.1, 1\}$; number of boosting rounds $p \in \{100, 500, 1000, 1500\}$; maximum tree depth for base learners $p \in \{3, 4, 5, 8, 10\}$; the subsample ratio of the training instance (selecting random training instances with higher probability when the gradient and hessian are larger) $p \in \{0, 0.5, 1\}$; the subsample ratio of features when constructing each tree $p \in \{0.2, 0.6, 0.8, 1.0\}$; minimum sum of instance weight (hessian) needed in a child $p \in \{1, 5, 10, 50, 100\}$; the ℓ_1 and ℓ_1 regularisation term on weights, $p \in \{10^{-5}, ..., 10^{-1}, ..., 0, ..., 10^0, 10^1, ..., 10^5\}$

List of Extracted Features

Supplementary Table 9. Description of the sensor-based features extracted in the weaRAble-PRO study

	Feature	Source	Dom.	Description
0	AvgBoutLen_MVPA	watch	AF	Avg. length of MVPA bouts
1	AvgBoutLen_light			Avg. length of active (light) bouts
2	AvgBoutLen_sedentary			Avg. length of sedentary bouts
3	AvgHazard_MVPAToany			Avg. hazard[1] of MVPA to non-MVPA
4	AvgHazard_anyToMVPA			Avg. hazard of non-MVPA to MVPA
5	AvgHazard_anyTosedentary			Avg. hazard of non-sedentary to sedentary
6	AvgHazard_sedentaryToany			Avg. hazard of sedentary to non-sedentary.
7	AvgLenTimeActive_MVPA			Avg. length of consecutive time in MVPA
8	AvgLenTimeActive_light			Avg. length of consecutive time active
9	AvgLenTimeActive_sedentary			Avg. length of consecutive time sedentary activity
10	BoutsHazard_MVPAToany			Avg. hazard of MVPA to non-MVPA bouts
11	BoutsHazard_anyToMVPA			Avg. hazard of non-MVPA to MVPA bouts
12	BoutsHazard_anyTosedentary			Avg. hazard of non-sedentary to sedentary bouts
13	BoutsHazard_sedentaryToany			Avg. hazard of sedentary to non-sedentary bouts
14	BoutsTransitionPr_MVPAToany			Transition probability of MVPA to non-MVPA bouts
15	BoutsTransitionPr_anyToMVPA			Transition probability of non-MVPA to MVPA bouts
16	BoutsTransitionPr_anyTosedentary			Transition probability of non-sedentary to sedentary bouts
17	BoutsTransitionPr_sedentaryToany			Transition probability of sedentary to non-sedentary bouts
18	RatioBoutsToActive			Ratio of time in active bouts to overall time active
19	TransitionPr_MVPAToany			Transition probability of active to sedentary (acc. intensity defined)
20	TransitionPr_anyToMVPA			Transition probability of active to sedentary (acc. intensity defined)
21	TransitionPr_anyTosedentary			Transition probability of active to sedentary (acc. intensity defined)
22	TransitionPr_sedentaryToany			Transition probability of active to sedentary (acc. intensity defined)
23	age_range		DEM	Age range [5 years]
24	sex			Sex [M/F]
25	LTS_mean_lie2stand	phone	LTS	Mean lie-to-stand transition time [s]
26	LTS_mean_lying			Mean lying time [s]
27	LTS_mean_stand2lie			Mean stand-to-lie transition time [s]
28	LTS_mean_standing			Mean standing time [s]
29	Morning stiffness: auc - 0:15:00	watch	MORN	Daily AUC of acc. vector magnitude during the first n=[15, 30, 45, 60, 120, 240] mins after getting up
30	Morning stiffness: auc - 0:30:00			
31	Morning stiffness: auc - 0:45:00			
32	Morning stiffness: auc - 1:00:00			
33	Morning stiffness: auc - 2:00:00			
34	Morning stiffness: auc - 4:00:00			
35	Morning stiffness: mean - 0:15:00			Daily avg. acc. vector magnitude during the first n=[15, 30, 45, 60, 120, 240] mins after getting up
36	Morning stiffness: mean - 0:30:00			
37	Morning stiffness: mean - 0:45:00			
38	Morning stiffness: mean - 1:00:00			
39	Morning stiffness: mean - 2:00:00			
40	Morning stiffness: mean - 4:00:00			
41	Morning stiffness: q(0.5) - 0:15:00			Daily median of acc. vector magnitude during the first n=[15, 30, 45, 60, 120, 240] mins after getting up
42	Morning stiffness: q(0.5) - 0:30:00			
43	Morning stiffness: q(0.5) - 0:45:00			
44	Morning stiffness: q(0.5) - 1:00:00			
45	Morning stiffness: q(0.5) - 2:00:00			
46	Morning stiffness: q(0.5) - 4:00:00			
47	Morning stiffness: q(0.95) - 0:15:00			Daily 95th centile of acc. vector magnitude during the first n=[15, 30, 45, 60, 120, 240] mins after getting up
48	Morning stiffness: q(0.95) - 0:30:00			
49	Morning stiffness: q(0.95) - 0:45:00			
50	Morning stiffness: q(0.95) - 1:00:00			
51	Morning stiffness: q(0.95) - 2:00:00			
52	Morning stiffness: q(0.95) - 4:00:00			
53	Morning stiffness: stdev - 0:15:00			Daily avg. of Standard deviation of acc. vector magnitude during the first n=[15, 30, 45, 60, 120, 240] mins after getting up
54	Morning stiffness: stdev - 0:30:00			
55	Morning stiffness: stdev - 0:45:00			
56	Morning stiffness: stdev - 1:00:00			
57	Morning stiffness: stdev - 2:00:00			
58	Morning stiffness: stdev - 4:00:00			
59	MSleep	watch	NTR	Daily avg. midpoint time of night-time sleep window [mins]
60	NSleepEpisodes			Avg. number of sleep episodes per night-time sleep window [count]
61	PercentSleep			Avg. percent time of sleep per night-time sleep window [%]
62	RestEfficiency			Avg. percent night-time rest efficiency per night-time sleep window
63	RestFragmentation			Avg. night-time rest fragmentation per night-time sleep window
64	SleepDur			Sleep duration [h]
65	SleepMov			The number of movement episodes per night-time sleep window [count]
66	SleepMovPerHr			The number of sleep movements per hour per night-time sleep window [count/hr]

Continued on Next Page…

[1] The average hazard reflects the probability for an individual to remain in a specified activity at minute t, or change to any other activity, given that the subject has been in a specified activity up to minute $t-1$[52].

Supplementary Table 9. Description of the sensor-based features extracted in the weaRAble-PRO study

	Feature	Source	Dom.	Description
67	TotDaySleep			The total amount of daytime sleep [mins]
68	PEG_totalFailures	phone	PEG	The total # of peg failures [count]
69	PEG_totalTime			The total 9HPT time [s]
70	STS_mean_sit2stand	phone	STS	Mean sit-to-stand transition time [s]
71	STS_mean_sitting			Mean sitting time [s]
72	STS_mean_stand2sit			Mean stand-to-sit transition time [s]
73	STS_mean_standing			Mean standing time [s]
74	95thCentileAccMag	watch	TVDA	The 95th centile of acc. vector magnitude [$m.s^{-2}$]
75	AvgAccInBout_MVPA			The study-avg. acc. in MVPA bouts [$m.s^{-2}$]
76	AvgAccInBout_light			The study-avg. acc. in light bouts [$m.s^{-2}$]
77	AvgAccInBout_sedentary			The study-avg. acc. in sedentary bouts [$m.s^{-2}$]
78	AvgAccMag			The study-avg. acc. vector magnitude [$m.s^{-2}$]
79	DailyAccInBout_MVPA			The daily acc. in MVPA bouts [$m.s^{-2}$]
80	DailyAccInBout_light			The daily acc. in light bouts [$m.s^{-2}$]
81	DailyAccInBout_sedentary			The daily acc. in sedentary bouts [$m.s^{-2}$]
82	DailyAvgAccMag			The daily avg. acc. vector magnitude [$m.s^{-2}$]
83	DailyAvgAcc_0-40mg			Daily avg. acc. in each 40 mg range [$m.s^{-2}$]
84	DailyAvgAcc_120-160mg			
85	DailyAvgAcc_160-200mg			
86	DailyAvgAcc_200-240mg			
87	DailyAvgAcc_240-280mg			
88	DailyAvgAcc_280-320mg			
89	DailyAvgAcc_320-360mg			
90	DailyAvgAcc_360-400mg			
91	DailyAvgAcc_40-80mg			
92	DailyAvgAcc_80-120mg			
93	DailyAvgAcc_MVPA			Daily avg. acc. during MVPA [$m.s^{-2}$]
94	DailyAvgAcc_light			Daily avg. acc. during sedentary [$m.s^{-2}$]
95	DailyAvgAcc_sedentary			Daily avg. acc. during MVPA [$m.s^{-2}$]
96	DailyAvgTimeInBout_MVPA			Daily avg. time spent in MVPA bouts [mins]
97	DailyAvgTimeInBout_light			Daily avg. time spent in light activity bouts [mins]
98	DailyAvgTimeInBout_sedentary			Daily avg. time spent sedentary bouts [mins]
99	DailyAvgTime_0-40mg			Daily avg. time spent in each 40 mg range [mins]
100	DailyAvgTime_120-160mg			
101	DailyAvgTime_160-200mg			
102	DailyAvgTime_200-240mg			
103	DailyAvgTime_240-280mg			
104	DailyAvgTime_280-320mg			
105	DailyAvgTime_320-360mg			
106	DailyAvgTime_360-400mg			
107	DailyAvgTime_40-80mg			
108	DailyAvgTime_80-120mg			
109	DailyAvgTime_MVPA			Daily avg. time spent in MVPA [mins]
110	DailyAvgTime_light			Daily avg. time spent in light activity [mins]
111	DailyAvgTime_sedentary			Daily avg. time spent in sedentary [mins]
112	DailyPctTimeInBout_MVPA			Daily percent of time spent in MVPA bouts [%]
113	DailyPctTimeInBout_light			Daily percent of time spent in light bouts [%]
114	DailyPctTimeInBout_sedentary			Daily percent of time spent sedentary bouts [%]
115	DailyPct_0-40mg			Daily percent of time in each 40 mg range [%]
116	DailyPct_120-160mg			
117	DailyPct_160-200mg			
118	DailyPct_200-240mg			
119	DailyPct_240-280mg			
120	DailyPct_280-320mg			
121	DailyPct_320-360mg			
122	DailyPct_360-400mg			
123	DailyPct_40-80mg			
124	DailyPct_80-120mg			
125	DailyPct_MVPA			Daily percent of time in MVPA [%]
126	DailyPct_Walking			Daily percent of time spent walking [%]
127	DailyPct_light			Daily percent of time in light activity [%]
128	DailyPct_sedentary			Daily percent of time spent sedentary [%]
129	MedianAccInBout_MVPA			Median acc. while in MVPA
130	MedianAccInBout_light			Median acc. while in light activity
131	MedianAccInBout_sedentary			Median acc. while in sedentary
132	MedianAccMag			Median acc. vector magntiude
133	PctTime_0-40mg	watch	TVDA	Study percent of time in each 40 mg range [%]
134	PctTime_120-160mg			
135	PctTime_160-200mg			
136	PctTime_200-240mg			
137	PctTime_240-280mg			
138	PctTime_280-320mg			
139	PctTime_320-360mg			
140	PctTime_360-400mg			
141	PctTime_40-80mg			

Continued on Next Page...

Supplementary Table 9. Description of the sensor-based features extracted in the weaRAble-PRO study

	Feature	Source	Dom.	Description
142	PctTime_80-120mg			
143	PctTime_MVPA			Study percent of time in MVPA [%]
144	PctTime_light			Study percent of time spent in light activity [%]
145	PctTime_sedentary			Study percent of time in sedentary activity [%]
146	PctTime_walking			Study percent of time spent walking [%]
147	StdAccMag			Standard deviation in acc. vector magnitude [m.s^{-2}]
148	nPeriods_Walking_120to600			Number of continuous walking periods with duration 2 to 10 minutes (with up to 30-second rest period) [count]
149	nPeriods_Walking_1800toinf			Number of continuous walking periods with duration >30 minutes (with up to 1-minute rest period) [count]
150	nPeriods_Walking_600to1800			Number of continuous walking periods with duration 10 to 30 minutes (with up to 1-minute rest period) [count]
151	WALK_numberOfSteps	phone	WLK	The daily number of steps [count]
152	WALK_stepFrequency			Cadence [steps/min]
153	WALK_stepLength			Step length [cm]
154	WALK_stepTime			Step time [sec]
155	WALK_stepVelocity			Walk velocity [m/s]
156	WRIST_ROM_global	phone	WRT	Range of motion (ROM) [deg]
157	WRIST_ROM_max			Maximum ROM [deg]
158	WRIST_ROM_mean			Mean ROM [deg]
159	WRIST_ROM_median			Median ROM [deg]
160	WRIST_ROM_min			Minimum ROM [deg]
161	WRIST_angvel_max			Maximum angular velocity [deg/s]
162	WRIST_angvel_mean			Mean angular velocity [deg/s]
163	WRIST_angvel_median			Median angular velocity [deg/s]
164	WRIST_angvel_min			Minimum angular velocity [deg/s]

References

1. Weir, J. P. Quantifying test-retest reliability using the intraclass correlation coefficient and the sem. *The J. Strength & Cond. Res.* **19**, 231–240 (2005).
2. Shrout, P. E. & Fleiss, J. L. Intraclass correlations: uses in assessing rater reliability. *Psychol. bulletin* **86**, 420 (1979).
3. Portney, L. G., Watkins, M. P. *et al.* *Foundations of clinical research: applications to practice*, vol. 892 (Pearson/Prentice Hall Upper Saddle River, NJ, 2009).
4. Beckerman, H. *et al.* Smallest real difference, a link between reproducibility and responsiveness. *Qual. Life Res.* **10**, 571–578 (2001).
5. Benjamini, Y. & Hochberg, Y. Controlling the false discovery rate: a practical and powerful approach to multiple testing. *J. Royal statistical society: series B (Methodological)* **57**, 289–300 (1995).
6. Hamy, V. *et al.* Patient-centric assessment of rheumatoid arthritis using a smartwatch and bespoke mobile app in a clinical setting. *Sci. Reports* **13**, 18311 (2023).
7. of Health, U. D. *et al.* Guidance for industry: patient-reported outcome measures: use in medical product development to support labeling claims: draft guidance. *Heal. Qual. Life Outcomes* **4**, 79 (2006).
8. Mercieca-Bebber, R., King, M. T., Calvert, M. J., Stockler, M. R. & Friedlander, M. The importance of patient-reported outcomes in clinical trials and strategies for future optimization. *Patient related outcome measures* **9**, 353 (2018).
9. Becker, B. *et al.* Development, psychometric evaluation and cognitive debriefing of the rheumatoid arthritis symptom and impact questionnaire (rasiq). *J. Patient-Reported Outcomes* **5**, 1–15 (2021).
10. Amtmann, D. *et al.* Development of a promis item bank to measure pain interference. *Pain* **150**, 173–182 (2010).
11. Buysse, D. J. *et al.* Development and validation of patient-reported outcome measures for sleep disturbance and sleep-related impairments. *Sleep* **33**, 781–792 (2010).
12. Maska, L., Anderson, J. & Michaud, K. Measures of functional status and quality of life in rheumatoid arthritis: health assessment questionnaire disability index (haq), modified health assessment questionnaire (mhaq), multidimensional health assessment questionnaire (mdhaq), health assessment questionnaire ii (haq-ii), improved health assessment questionnaire (improved haq), and rheumatoid arthritis quality of life (raqol). *Arthritis care & research* **63**, S4–S13 (2011).
13. Hewlett, S., Dures, E. & Almeida, C. Measures of fatigue: Bristol rheumatoid arthritis fatigue multi-dimensional questionnaire (braf mdq), bristol rheumatoid arthritis fatigue numerical rating scales (braf nrs) for severity, effect, and coping, chalder fatigue questionnaire (cfq), checklist individual strength (cis20r and cis8r), fatigue severity scale (fss), functional assessment chronic illness therapy (fatigue)(facit-f), multi-dimensional assessment of fatigue (maf), multi-dimensional fatigue inventory (mfi), pediatric quality of life (pedsql) multi-dimensional fatigue scale, profile of fatigue (prof), short form 36 vitality subscale (sf-36 vt), and visual analog scales (vas). *Arthritis care & research* **63**, S263–S286 (2011).
14. Cella, D. *et al.* Validation of the functional assessment of chronic illness therapy fatigue scale relative to other instrumentation in patients with rheumatoid arthritis. *The J. rheumatology* **32**, 811–819 (2005).
15. Hewlett, S., Hehir, M. & Kirwan, J. R. Measuring fatigue in rheumatoid arthritis: a systematic review of scales in use. *Arthritis Care & Res.* **57**, 429–439 (2007).
16. Ware Jr, J. E. Sf-36 health survey update. *Spine* **25**, 3130–3139 (2000).
17. ten Klooster, P. M. *et al.* Performance of the dutch sf-36 version 2 as a measure of health-related quality of life in patients with rheumatoid arthritis. *Heal. quality life outcomes* **11**, 1–9 (2013).
18. Matcham, F. *et al.* The impact of rheumatoid arthritis on quality-of-life assessed using the sf-36: a systematic review and meta-analysis. In *Seminars in arthritis and rheumatism*, vol. 44, 123–130 (Elsevier, 2014).
19. Hamy, V. *et al.* Developing smartphone-based objective assessments of physical function in rheumatoid arthritis patients: the PARADE study. *Digit. biomarkers* **4**, 26–44 (2020).
20. Langley, G. & Sheppeard, H. The visual analogue scale: its use in pain measurement. *Rheumatol. international* **5**, 145–148 (1985).
21. Yuan, H. *et al.* Self-supervised learning for human activity recognition using 700,000 person-days of wearable data. *arXiv preprint arXiv:2206.02909* (2022).
22. He, K., Zhang, X., Ren, S. & Sun, J. Identity mappings in deep residual networks. In *European conference on computer vision*, 630–645 (Springer, 2016).

23. Ainsworth, B. E. *et al.* 2011 compendium of physical activities: a second update of codes and met values. *Med Sci Sports Exerc.* **43**, 1575–1581 (2011).

24. Walmsley, R. *et al.* Reallocating time from device-measured sleep, sedentary behaviour or light physical activity to moderate-to-vigorous physical activity is associated with lower cardiovascular disease risk. *MedRxiv* (2020).

25. Willetts, M., Hollowell, S., Aslett, L., Holmes, C. & Doherty, A. Statistical machine learning of sleep and physical activity phenotypes from sensor data in 96,220 uk biobank participants. *Sci. reports* **8**, 1–10 (2018).

26. Prioreschi, A., Hodkinson, B., Avidon, I., Tikly, M. & McVeigh, J. A. The clinical utility of accelerometry in patients with rheumatoid arthritis. *Rheumatology* **52**, 1721–1727 (2013).

27. Henchoz, Y. *et al.* Physical activity and energy expenditure in rheumatoid arthritis patients and matched controls. *Rheumatology* **51**, 1500–1507 (2012).

28. Grassi, W., De Angelis, R., Lamanna, G. & Cervini, C. The clinical features of rheumatoid arthritis. *Eur. journal radiology* **27**, S18–S24 (1998).

29. Harkness, J. *et al.* Circadian variation in disease activity in rheumatoid arthritis. *Br Med J (Clin Res Ed)* **284**, 551–554 (1982).

30. Earhart, G. M. *et al.* The 9-hole peg test of upper extremity function: average values, test-retest reliability, and factors contributing to performance in people with parkinson disease. *J. Neurol. Phys. Ther.* **35**, 157–163 (2011).

31. Crouthamel, M. *et al.* Using a researchkit smartphone app to collect rheumatoid arthritis symptoms from real-world participants: feasibility study. *JMIR mHealth uHealth* **6**, e9656 (2018).

32. Zijlstra, W. & Hof, A. L. Displacement of the pelvis during human walking: experimental data and model predictions. *Gait & posture* **6**, 249–262 (1997).

33. Godfrey, A., Del Din, S., Barry, G., Mathers, J. & Rochester, L. Instrumenting gait with an accelerometer: a system and algorithm examination. *Med. engineering & physics* **37**, 400–407 (2015).

34. Zhao, H. *et al.* Smartphone-based 3d indoor pedestrian positioning through multi-modal data fusion. *Sensors* **19**, 4554 (2019).

35. Andreu-Perez, J. *et al.* Developing fine-grained actigraphies for rheumatoid arthritis patients from a single accelerometer using machine learning. *Sensors* **17**, 2113 (2017).

36. Schwickert, L. *et al.* Inertial sensor based analysis of lie-to-stand transfers in younger and older adults. *Sensors* **16**, 1277 (2016).

37. Mathiowetz, V., Weber, K., Kashman, N. & Volland, G. Adult norms for the nine hole peg test of finger dexterity. *The Occup. Ther. J. Res.* **5**, 24–38 (1985).

38. Bohannon, R. W. Sit-to-stand test for measuring performance of lower extremity muscles. *Percept. motor skills* **80**, 163–166 (1995).

39. Little, R. J. & Rubin, D. B. Statistical analysis with missing data. john wiley & sons. *New York* (2002).

40. Sakia, R. M. The box-cox transformation technique: a review. *J. Royal Stat. Soc. Ser. D (The Stat.* **41**, 169–178 (1992).

41. Rao, C. R. *Linear statistical inference and its applications*, vol. 2 (Wiley New York, 1973).

42. Hastie, T., Tibshirani, R. & Friedman, J. *The elements of statistical learning: data mining, inference, and prediction* (Springer Science & Business Media, 2009).

43. McCullagh, P. *Generalized linear models* (Routledge, 2018).

44. Hosmer Jr, D. W., Lemeshow, S. & Sturdivant, R. X. *Applied logistic regression*, vol. 398 (John Wiley & Sons, 2013).

45. Hastie, T., Tibshirani, R. & Wainwright, M. *Statistical learning with sparsity: the lasso and generalizations* (CRC press, 2015).

46. Tibshirani, R. Regression shrinkage and selection via the lasso. *J. Royal Stat. Soc. Ser. B (Methodological)* 267–288 (1996).

47. Simon, N., Friedman, J., Hastie, T. & Tibshirani, R. A sparse-group lasso. *J. computational graphical statistics* **22**, 231–245 (2013).

48. Breiman, L. Random forests. *Mach. learning* **45**, 5–32 (2001).

49. Chen, T. & Guestrin, C. Xgboost: A scalable tree boosting system. In *Proceedings of the 22nd acm sigkdd international conference on knowledge discovery and data mining*, 785–794 (2016).

50. Strobl, C., Boulesteix, A.-L., Kneib, T., Augustin, T. & Zeileis, A. Conditional variable importance for random forests. *BMC bioinformatics* **9**, 307 (2008).

51. Genuer, R., Poggi, J.-M. & Tuleau-Malot, C. Variable selection using random forests. *Pattern recognition letters* **31**, 2225–2236 (2010).

52. Di, J. *et al.* Patterns of sedentary and active time accumulation are associated with mortality in us adults: The nhanes study. *bioRxiv* 182337 (2017).
